# Supplementary material for: Structural and functional analysis of the promiscuous AcrB and AdeB efflux pumps suggests different drug binding mechanisms
Source: Nat Commun. 2021 Nov 25;12:6919. doi: 10.1038/s41467-021-27146-2 (PMC8617272; doi:10.1038/s41467-021-27146-2)
Supplement: Supplementary file 1 — Supplementary Information [file 41467_2021_27146_MOESM1_ESM.pdf]

Supplementary Information

Structural and functional analysis of the promiscuous AcrB and AdeB efflux pumps suggests different drug binding mechanisms

Alina Ornik-Cha[1§], Julia Wilhelm[1§], Jessica Kobylka[1], Hanno Sjuts[1,2], Attilio V. Vargiu[3], Giuliano Malloci[3], Julian Reitz[4,5], Anja Seybert[4,5], Achilleas S. Frangakis[4,5*], Klaas M. Pos[1*]

[1]Institute of Biochemistry, Goethe-University Frankfurt, Max-von-Laue-Str. 9, D-60438 Frankfurt am Main, Germany.

[2]present address: Biologics Research, Sanofi-Aventis Deutschland GmbH, Frankfurt, Germany

[3]Department of Physics, University of Cagliari, 09042 Monserrato (CA), Italy

[4]Buchmann Institute for Molecular Life Sciences, Goethe-University Frankfurt, Max-von-Laue-Str. 15, D-60438 Frankfurt am Main, Germany.

[5]Institute of Biophysics, Goethe-University Frankfurt, Max-von-Laue-Str. 15, D-60438 Frankfurt am Main, Germany.

[§]Contributed equally

* Correspondence to Klaas M. Pos (pos@em.uni-frankfurt.de) or Achilleas Frangakis (achilleas.frangakis@biophysik.org.)

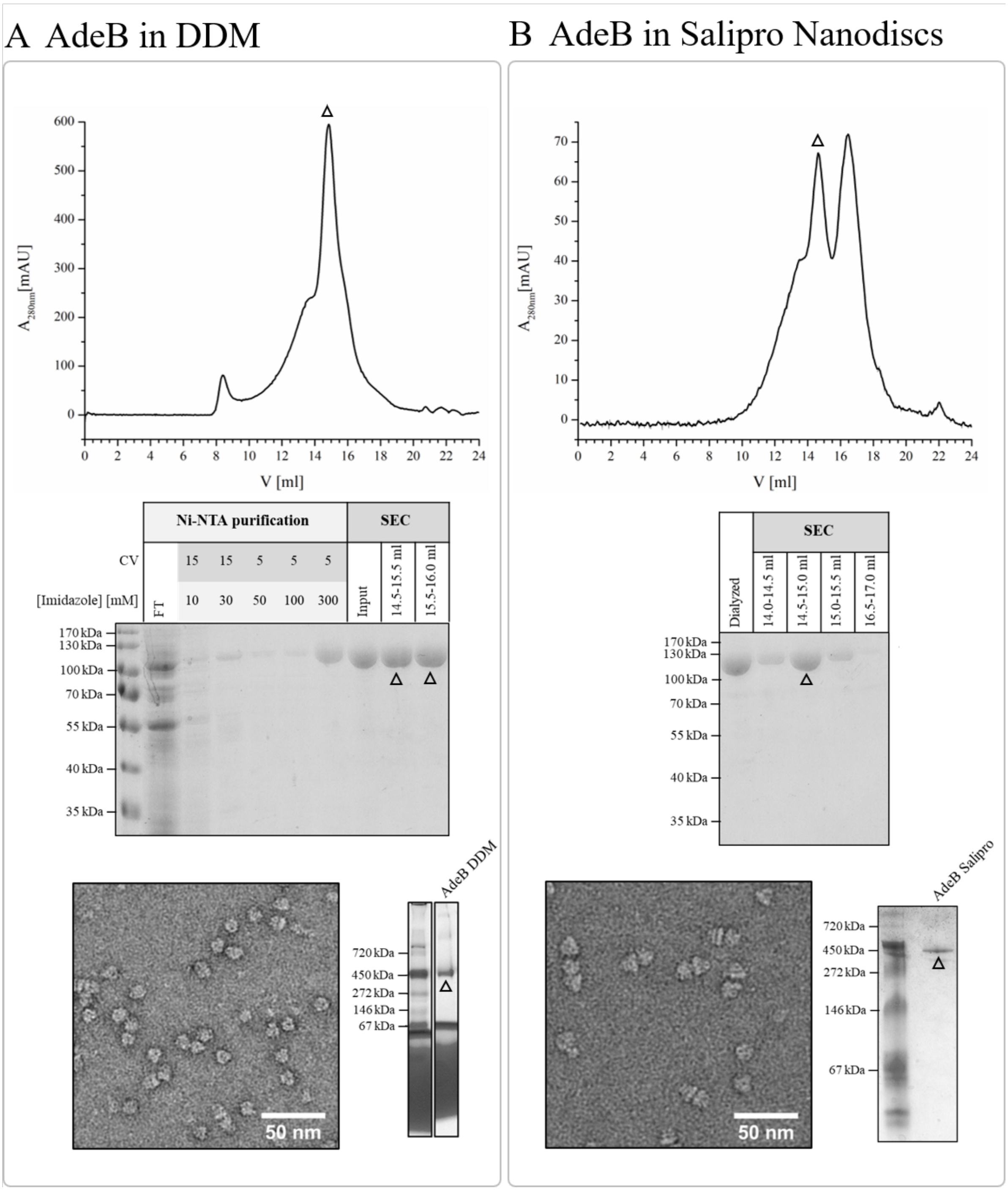

Supplementary Figure 1. **Purification and reconstitution of AdeB into Salipro Nanodiscs. A** AdeB was purified after heterologous expression in *E. coli* by Ni-NTA IMAC followed by size exclusion chromatography (SEC). Samples were analyzed by SDS-PAGE showing high purity. A homogenous trimeric assembly could be confirmed by negative stain EM and native PAGE. **B** AdeB was reconstituted in Salipro Nanodiscs and purified by SEC. Again, samples were analyzed by SDS-PAGE, negative stain EM and native PAGE showing high purity of the trimeric particles. The fractions selected for further procedures are marked by triangles. SEC and SDS-PAGE analysis was conducted three times and Native PAGE/negative stain EM was done once. Source data are provided as a Source Data file.

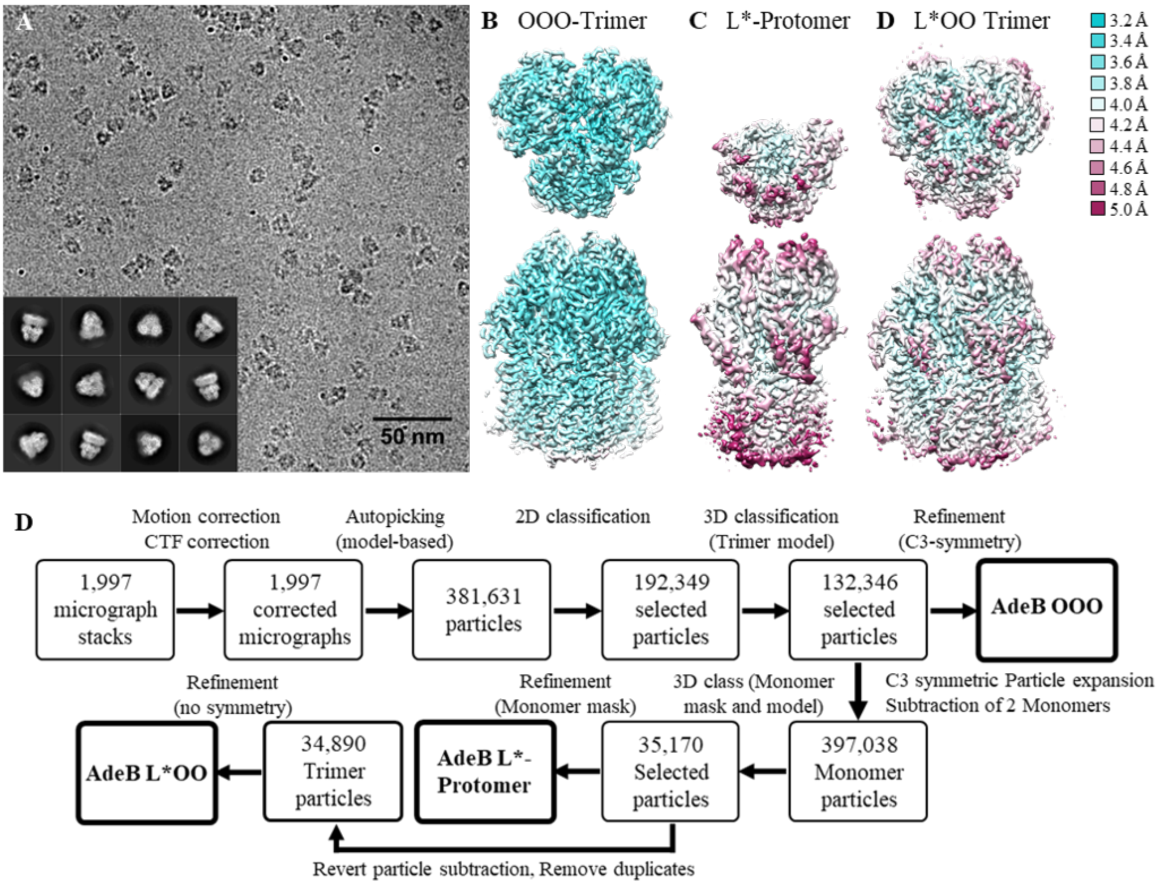

Supplementary Figure 2. **Cryo-EM density maps of AdeB. A** Exemplary micrograph (out of 1,997 micrographs, supplementary Table 1) of AdeB at -3.7 µm defocus and representative 2D class averages. Density maps **B**, **C**, **D** are shown in top and side view and colored by local resolution. **B** AdeB density map in OOO conformation with an overall resolution of 3.54 Å. **C** AdeB L* conformation with a resolution of 3.95 Å. **D** AdeB in L*OO conformation at 3.84 Å resolution. The L* conformation is oriented to the front. **E** Flowchart of the data processing procedure. FSC curves and angular distribution of all maps are shown in Supplementary Figure 3.

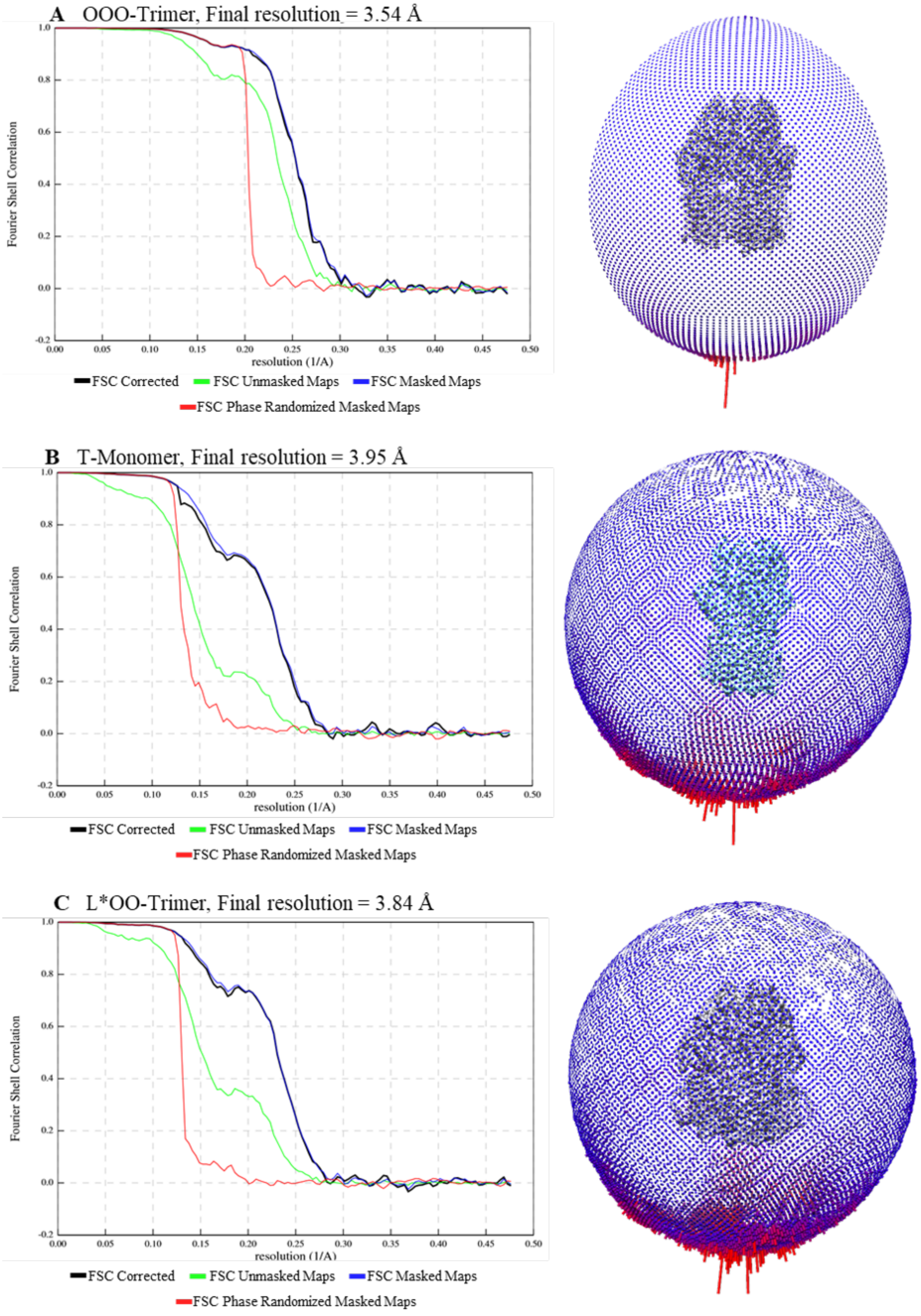

Supplementary Figure 3. **Fourier shell correlation (FSC) and angular distribution of AdeB density maps**. **A** OOO conformation, **B** L* conformation and **C** L*OO conformation.

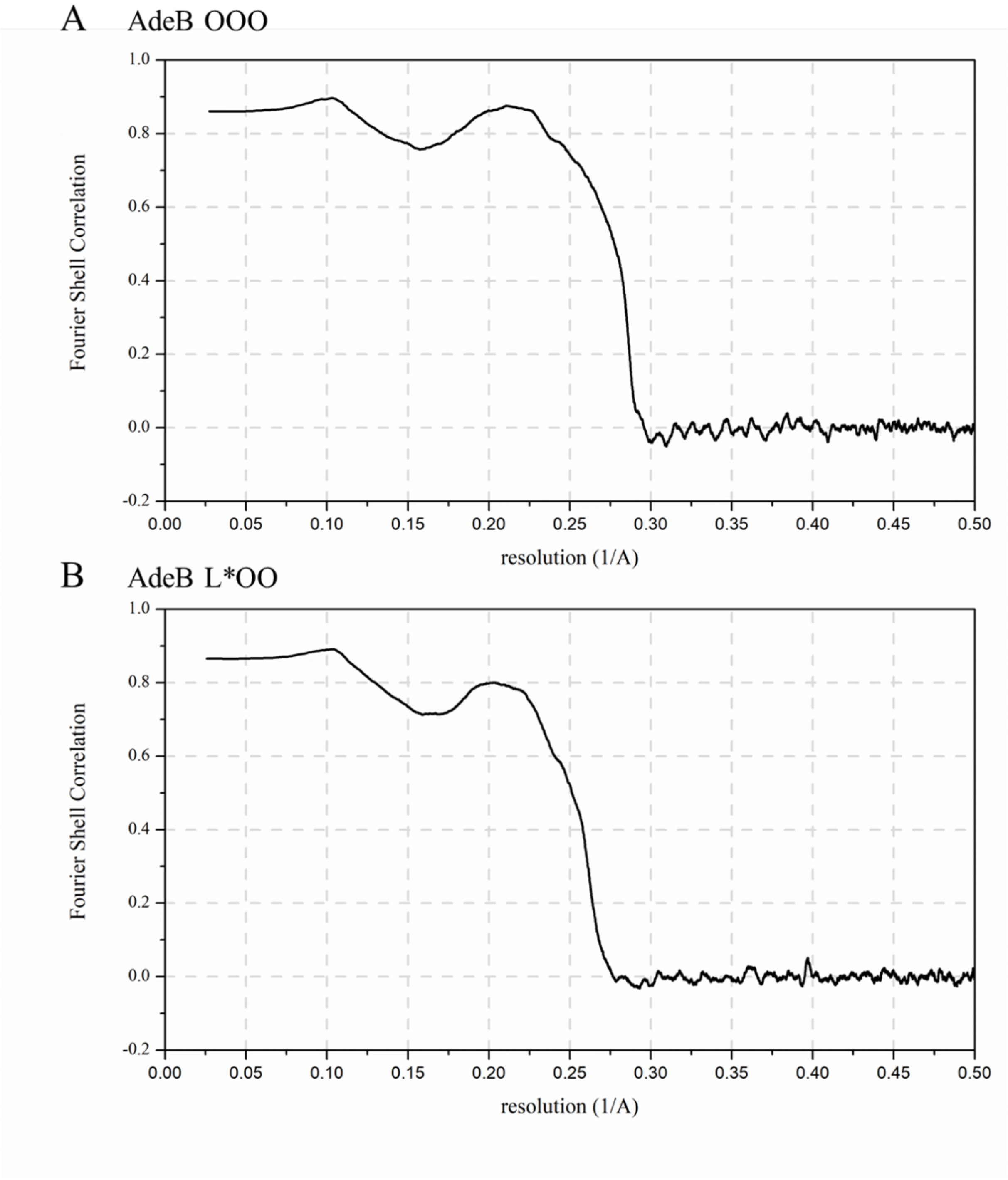

Supplementary Figure 4. **Fourier shell correlation (FSC) of EM maps to modelled structures of AdeB.** **A** OOO and **B** L*OO conformations.

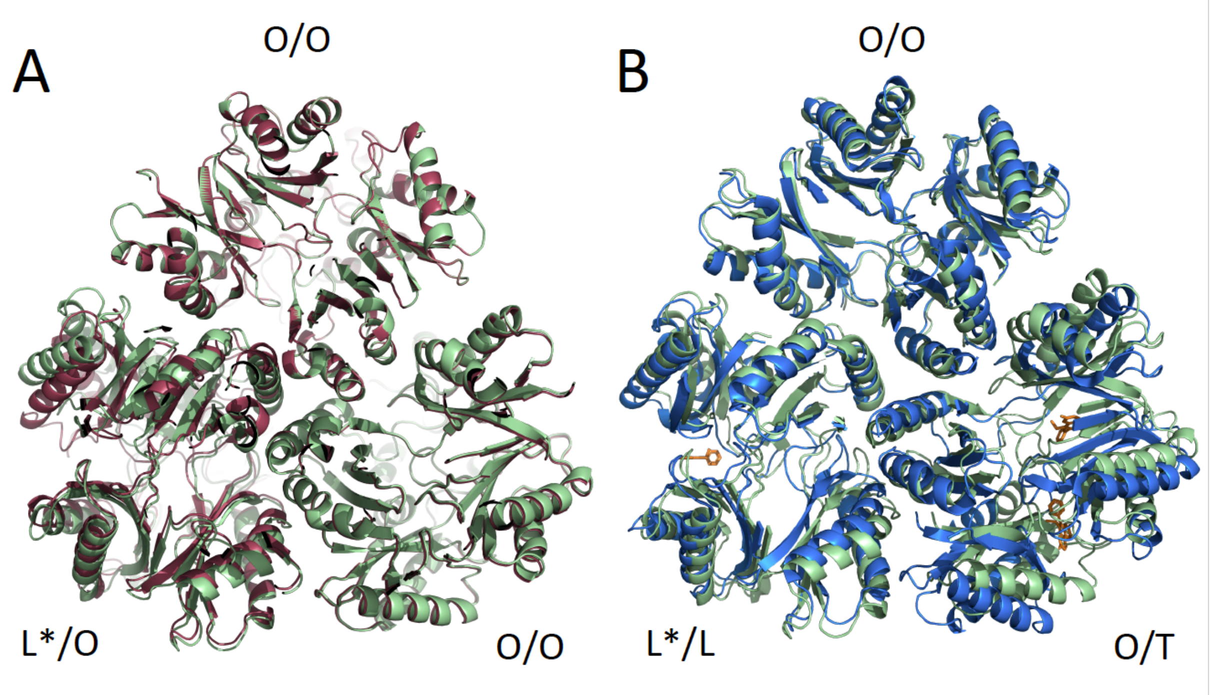

Supplementary Figure 5. **Superimposition of the porter domain of the L*OO structure (green) with (A) the OOO structure (red) and (B) LTO (access/binding/extrusion, PDB: 7KGI) structure (blue).** Ethidium molecules bound to the L and T protomers in the LTO structure are displayed as yelloworange sticks.

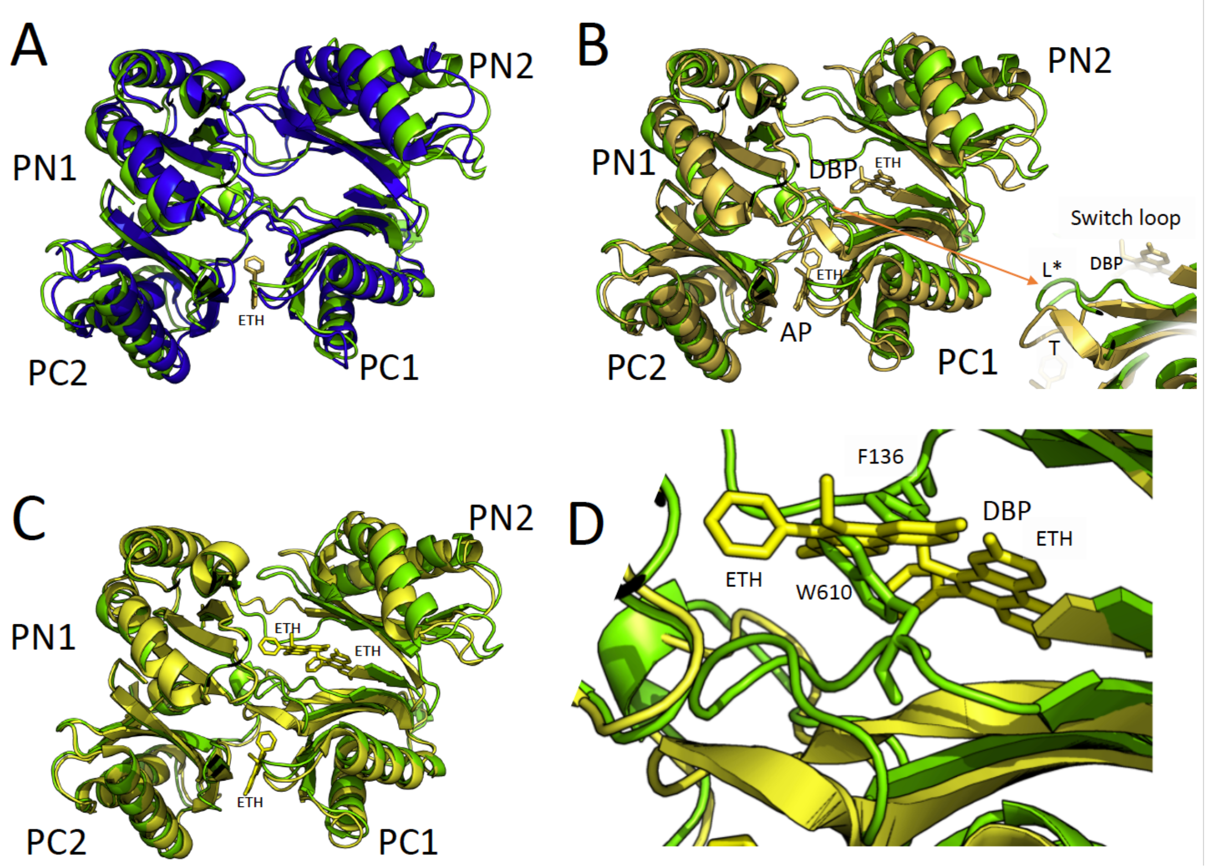

Supplementary Figure 6. **Superimpositions of the AdeB periplasmic porter domains.** Superimposition of the L* protomer (green) with **A** the L (access, blue) conformation of the LTO structure (access/binding/extrusion, PDB: 7KGI) with ethidum (yellow sticks) bound in the access pocket (AP) (rmsd: 2,1 A), with **B** the T conformation (orangeyellow) of the LTO structure (access/binding/extrusion, PDB: 7KGI) with ethidum (yellow sticks) bound in the AP and the deep binding pocket (DBP) (rmsd: 1,9 A). Inset: the L* switch loop (green) is oriented toward the DBP, whereas the T switch loop (yellow) orientation is toward the AP. **C** the T conformation (yellow) of the TOO structure (binding/extrusion/extrusion, PDB: 7KGH) with ethidum (yellow sticks) bound in the AP and two ethidium molecules (yellow sticks) bound to the DBP (rmsd: 1,6 A). **D** residues F136 and W610 (green sticks) in the L* protomer are given as an example to indcate that substrate binding in this protomer is prohibited in the DBP due to steric clash.

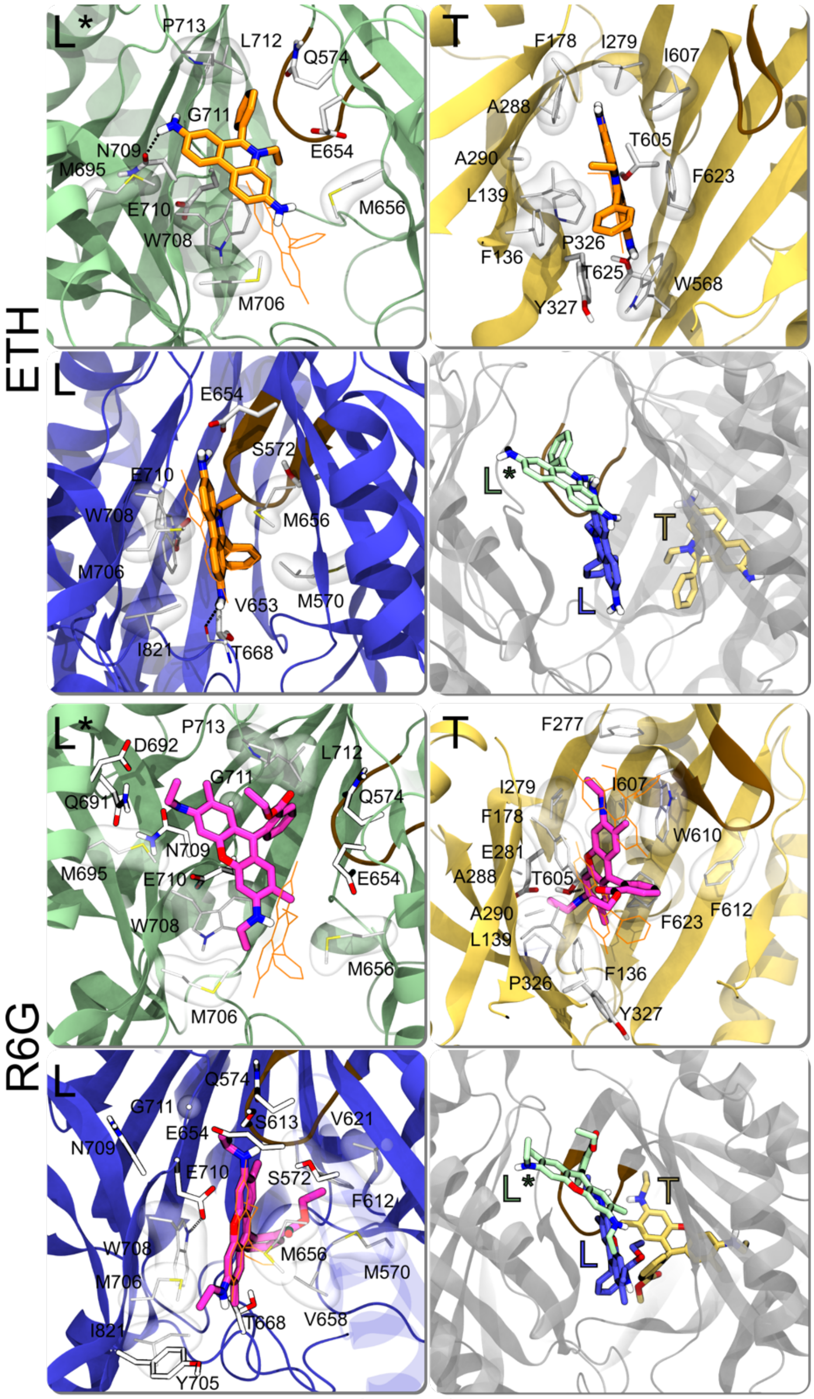

State	ΔG [kcal/mol] L*	L	T
ETH	-19.1	-31.3	-38.7
R6G	-31.5	-17.5	-43.1

Supplementary Figure 7. **Selected docking poses of ETH and R6G on the L*, L, and T protomers of AdeB.** Docking poses of ETH (top 2x2 panel, C atoms in orange) and R6G (bottom panel, C atoms in magenta) in the L*, L, and T protomers (cartoon in green, blue, and yellow color, respectively). The switch loop is highlighted in ochre. Sidechains (or backbone atoms forming H-bonds with the ligand) of polar and apolar residues within 3.5 Å of the ligand are shown by thick and thin sticks, respectively, colored by atom type (C atoms in light grey). The approximate molecular envelope of apolar residues is also shown as transparent surface. The experimental poses of ETH in the L and T protomers (PDB IDs: 7KGI and 7KGG) are shown as reference (thin orange sticks). The bottom-right picture in each panel shows the combined docking poses in the L*, L and T protomers as sticks colored by protomer (L*, green, L, blue, and T, yellow). Below the images the estimated ΔG values for ETH and R6G binding are listed.

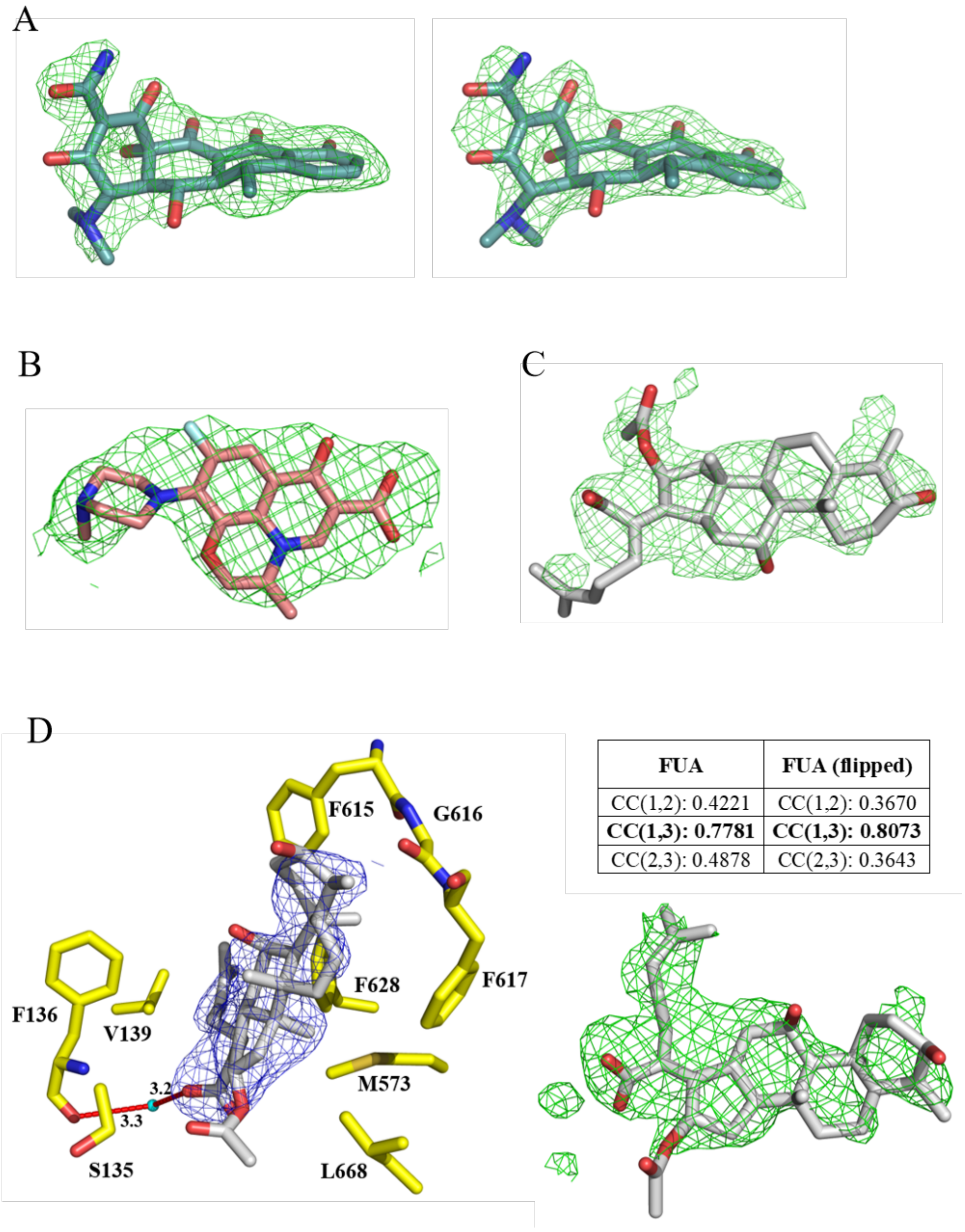

FUA	FUA (flipped)
CC(1,2): 0.4221	CC(1,2): 0.3670
**CC(1,3): 0.7781**	**CC(1,3): 0.8073**
CC(2,3): 0.4878	CC(2,3): 0.3643

Supplementary Figure 8. **Residual (Polder) electron density maps.** Polder maps of **A** doxycycline (DXT-1, left; DXT-2, right), **B** levofloxacin (LFX) and **C** fusidic acid (FUA). Polder maps (green-colored mesh) are contoured at 4.5σ (DXT) or 4σ (LFX, FUA). The assigned ligand molecules are represented as sticks (carbon = dark green (DXT); carbon = salmon (LFX); carbon = grey (FUA); nitrogen = blue; oxygen = red; fluoride = pale blue). **D** Alternative (flipped) FUA orientation within the AcrBper DBP. The $2F_o$-F_c electron density map (blue-colored mesh) is contoured at 0.8σ and the residual (Polder) electron density map is contoured at 4σ. Local correlation coefficients (CC) between three Polder maps m1 (calculated F_{obs} with ligand), m2 (calculated F_{obs} without ligand) and m3 (real F_{obs} data) are indicated for both FUA conformations.

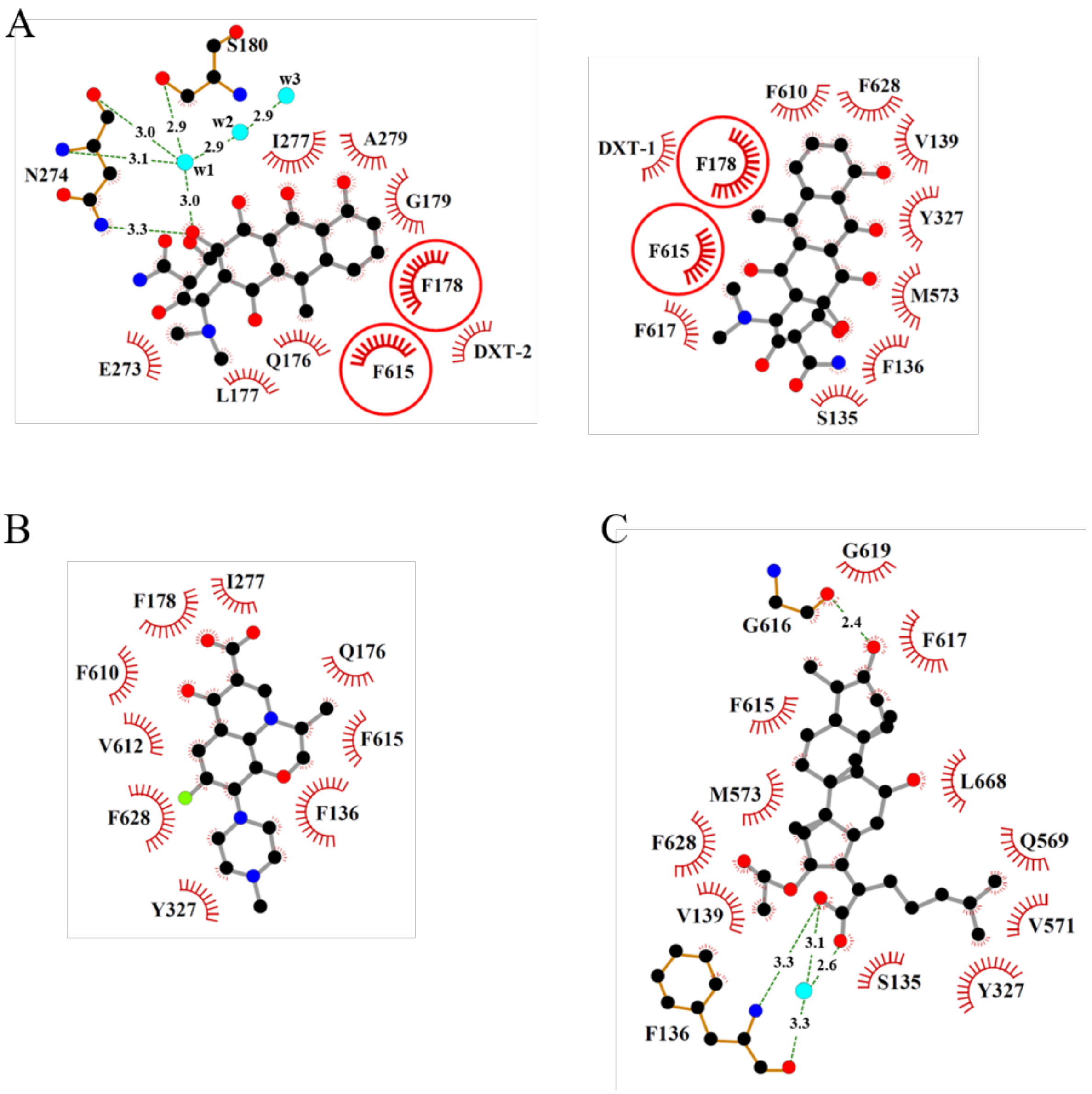

Supplementary Figure 9. **LigPlot+ analysis of ligand binding to the AcrBper DBP.** **A** LigPlot+ analysis of the two doxycycline binding modes, DXT (left) and DXT-2 (right), to the AcrBper DBP. AcrBper residues interacting with both DXT molecules are circled in red. **B** LigPlot+ analysis of levofloxacin and **C** fusidic acid binding to the AcrBper DBP. The ligands are shown in ball-and-stick representation (carbon = black; nitrogen = blue; oxygen = red; fluoride = green; bonds = grey). Hydrophobic interactions between the ligands and AcrBper DBP residues are represented with red brush-like structures. Water molecules and hydrogen bonds are shown as cyan spheres and green dashed lines, respectively, with the numbers representing the H-bond distances in Å.

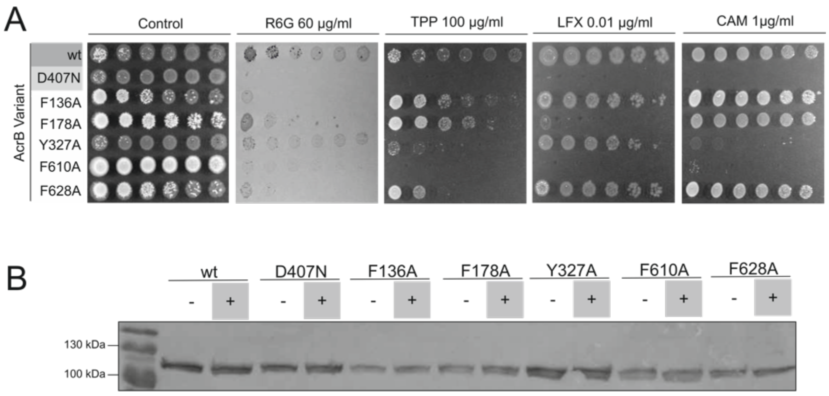

Supplementary Figure 10. **Comparison of drug susceptibilities of *E. coli* cells harbouring *E. coli* AcrB, wildtype, inactive mutant (D407N) and deep binding pocket AcrB single-substitution variants (F136A, F178A, Y327A, F610A, F628A).** **A** Plate dilution assays were performed with *E. coli* BW25113 Δ*acrB* Δ*acrD* Δ*mdtBC* pRSFDuetFX_MS_*adeAC* harbouring pET24_acrB or mutants. Dilution series of overnight cultures with an OD_{600} of 10^0, 10^{-1}, 10^{-2}, 10^{-3}, 10^{-4} and 10^{-5} were spotted on a Mueller-Hinton (MH) agar plates containing 50 µg/ml kanamycin (Km), 50 µg/ml carbenicillin (Carb), 20 µM IPTG with or without (control plates) the tested drug. Plates were supplemented with the compounds and concentrations indicated. All experiments were performed four times (biological replicates 1-4, on different days with newly transformed clones), a representative experiment is shown. **B** Western blot analysis of DDM-solubilized protein samples before (-) and after (+) ultracentrifugation to detect levels of correctly folded AcrB variants via anti-AcrB antibody. Western Blot analysis was done once. Plate dilution biological replicate results 1-4 and Western Blot analysis full scan image is available in a Source Data file.

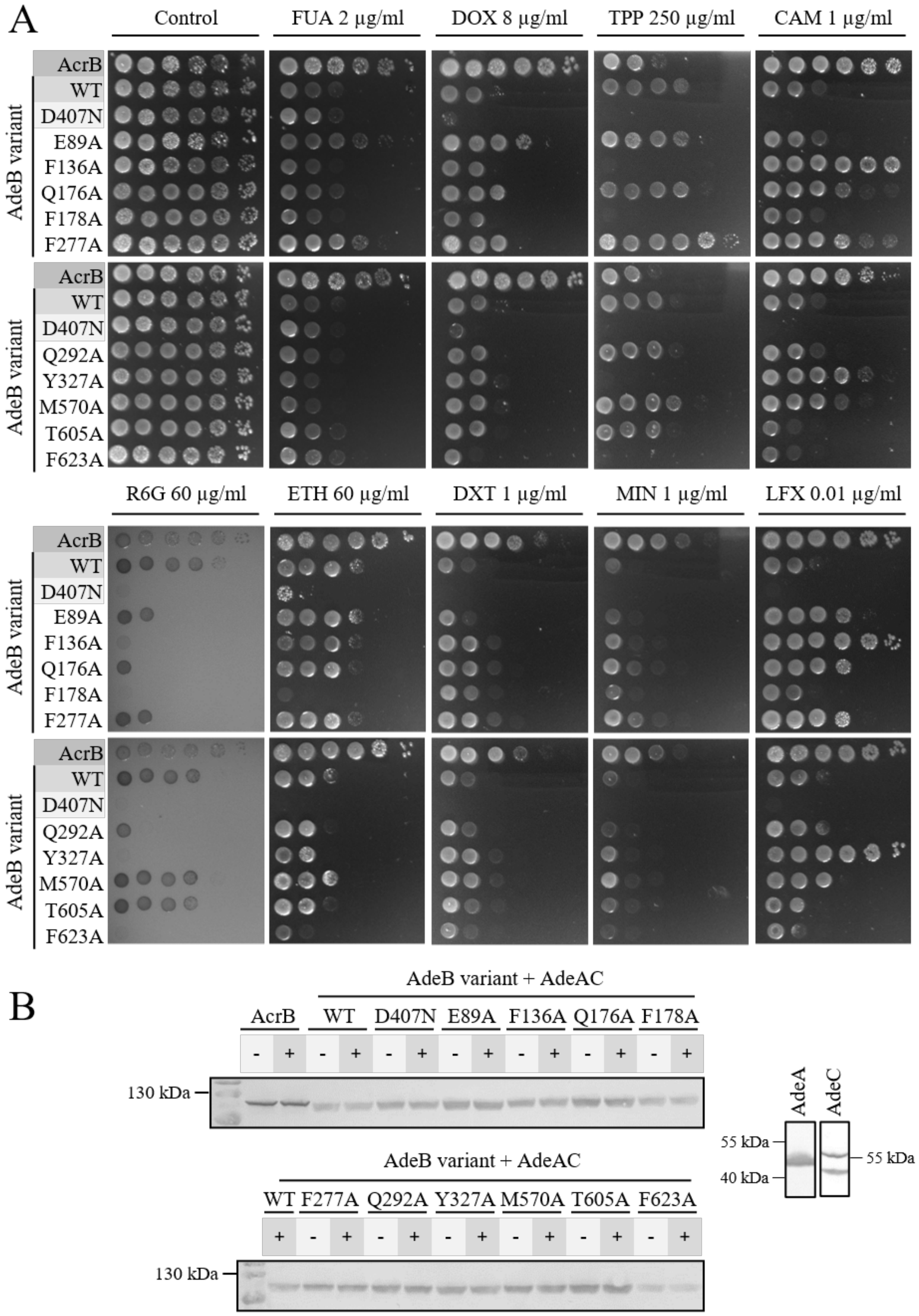

Supplementary Figure 11. **Comparison of drug susceptibilities of *E. coli* cells harbouring *E. coli* AcrB, wildtype *A. baumannii* AdeB (WT), inactive mutant (D407N) and deep binding pocket single-Ala variants (E89A, F136A, Q176A, F178A, F277A, Q292A, Y327A, M570A, T605A, F623A). A** Plate dilution assays were performed with *E. coli* BW25113 *ΔacrB ΔacrD ΔmdtBC* pRSFDuetFX_MS_*adeAC* harboring pET24_*acrB* or p7XC3H_*adeB_WT* and mutants. Dilution series of overnight cultures with an OD600 of 10^0, 10^{-1}, 10^{-2}, 10^{-3}, 10^{-4} and 10^{-5} were spotted on a Mueller-Hinton Agar plate containing 50 µg/ml kanamycin, 50 µg/ml carbenicillin, 20 µM IPTG with or without (control plate) the tested drug. Plates were supplemented with the following compounds: 2 µg/ml fusidic acid (FUA), 8 µg/ml doxorubicin (DOX), 250 µg/ml TPP, 1 µg/ml chloramphenicol (CAM), 60 µg/ml rhodamine-6G (R6G), 60 µg/ml ethidium (ETH), 1 µg/ml doxycycline (DXT), 1 µg/ml minocycline (MIN), and 0.01 µg/ml levofloxacin (LFX). All experiments were performed in triplicate. **B** Western blot analysis of DDM-solubilized protein samples before (-) and after (+) ultracentrifugation to detect levels of correctly folded AcrB, AdeB WT and variants via His-tag. Western Blot analysis has been done in triplicate, twice with whole cell lysates and once with DDM-solubilized protein samples as shown here. All results and uncropped images are available in Source Data.

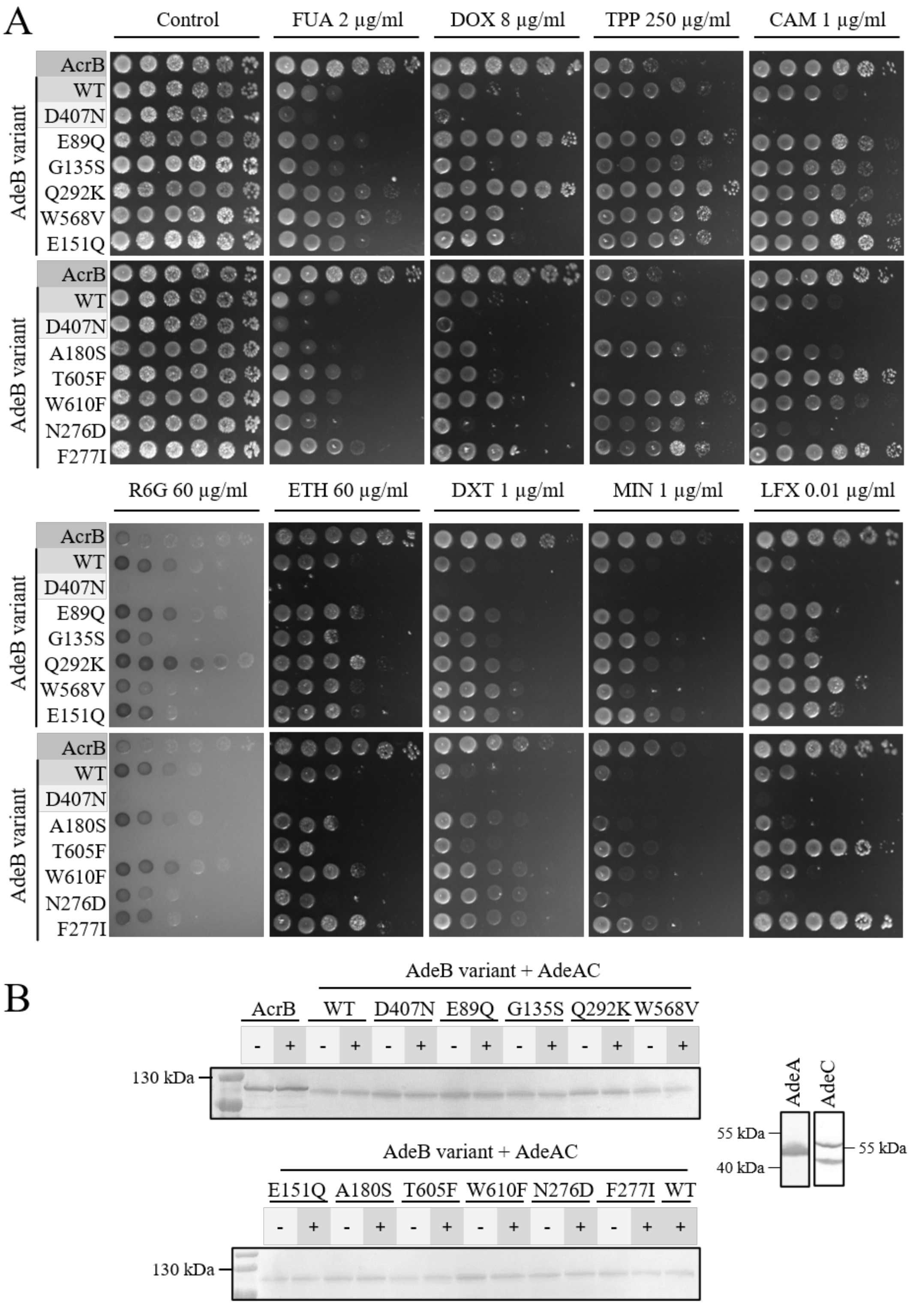

Supplementary Figure 12. **Comparison of drug susceptibilities of *E. coli* cells harbouring *E. coli* AcrB, wildtype *A. baumannii* AdeB (WT), inactive mutant (D407N) and deep binding pocket AdeB to AcrB single-substitution variants (E89Q, G135S, Q292K, W568V, E151Q, A180S, T605F, W610F, N276D, F277I).** **A** Plate dilution assays were performed with *E. coli* BW25113 *ΔacrB ΔacrD ΔmdtBC* pRSFDuetFX_MS_*adeAC* harbouring pET24_*acrB* or p7XC3H_*adeB_WT* and mutants. Dilution series of overnight cultures with an OD600 of 10^{0}, 10^{-1}, 10^{-2}, 10^{-3}, 10^{-4} and 10^{-5} were spotted on a Mueller-Hinton Agar plate containing 50 µg/ml kanamycin, 50 µg/ml carbenicillin, 20 µM IPTG with or without (control plate) the tested drug. Plates were supplemented with the following compounds: 2 µg/ml fusidic acid (FUA), 8 µg/ml doxorubicin (DOX), 250 µg/ml TPP, 1 µg/ml chloramphenicol (CAM), 60 µg/ml rhodamine-6G (R6G), 60 µg/ml ethidium (ETH), 1 µg/ml doxycycline (DXT), 1 µg/ml minocycline (MIN), and 0.01 µg/ml levofloxacin (LFX). All experiments were performed in triplicate. **B** Western blot analysis of DDM-solubilized protein samples before (-) and after (+) ultracentrifugation to detect levels of correctly folded AcrB, AdeB WT and variants via His-tag. Western Blot analysis has been done in triplicate, twice with whole cell lysates and once with DDM-solubilized protein samples as shown here. All results and uncropped images are provided in a Source Data file.

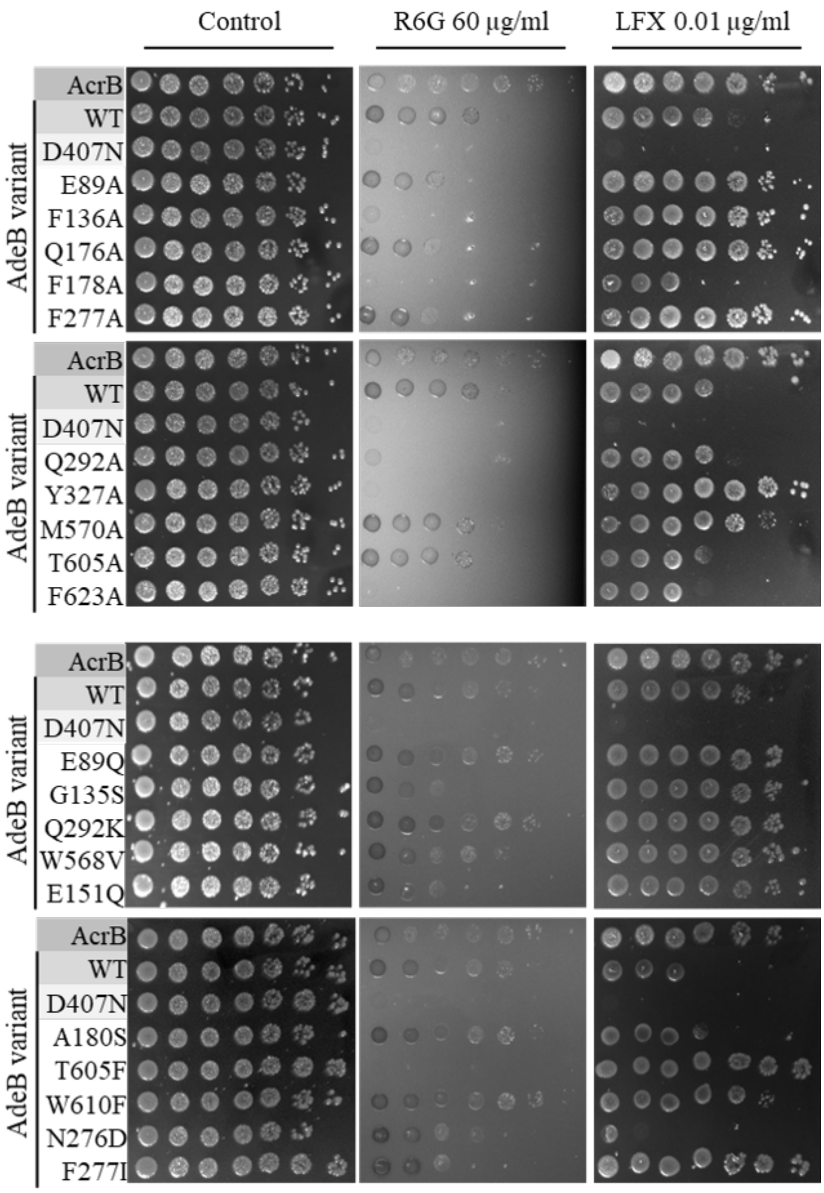

Supplementary Figure 13. **Comparison of drug susceptibilities of *E. coli* cells harbouring *E. coli* AcrB, wildtype *A. baumannii* AdeB (WT), inactive mutant (D407N) and deep binding pocket variants (E89A, F136A, Q176A, F178A, F277A, Q292A, Y327A, M570A, T605A, F623A, E89Q, G135S, Q292K, W568V, E151Q, A180S, T605F, W610F, N276D, F277I).** Plate dilution assays were performed with *E. coli* BW25113 *ΔacrB ΔacrD ΔmdtBC* pRSFD_*adeAC* harbouring pET24_*acrB* or p7XC3H_*adeB_WT* or mutants. Dilution series of overnight cultures with an OD_{600} of 10^0, 10^{-1}, 10^{-2}, 10^{-3}, 10^{-4} and 10^{-5} were spotted on a Mueller-Hinton Agar plate containing 20 µM IPTG (w/o additional antibiotics) with or without (control plate) the tested drug. Plates were supplemented with 60 µg/ml rhodamine-6G (R6G) or 0.01 µg/ml levofloxacin (LFX). All experiments were performed in triplicate and source data are provided as a Source Data file.

A

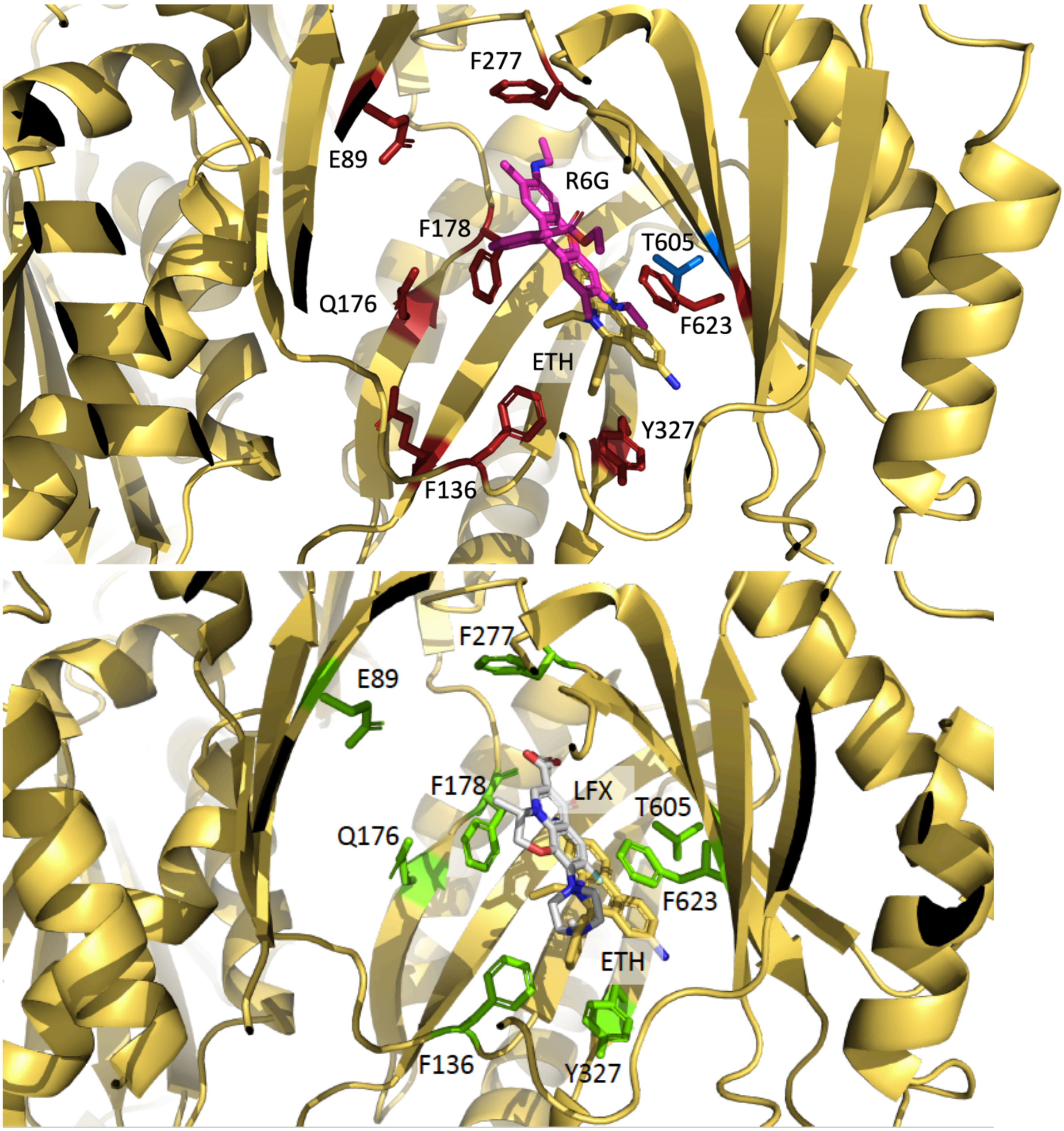

B

Supplementary Figure 14. **Superimpositions of drug binding in the deep binding pocket (DBP) of AcrB and AdeB.** The AdeB T protomer DBP region is displayed as yelloworange cartoon. Superimposition of **A** AcrBper with rhodamine 6G (R6G, magenta) bound (PDB: 5ENS). Residues which are negatively affecting the susceptibilities for R6G after substitution with Ala are indicated as red sticks. T605 is indicated as blue stick. **B** AcrBper with levofloxacin (LFX, grey) bound (PDB: 7B8T, this work) to the AdeB T protomer with ethidium (ETH, yelloworange) bound (PDB: 7KGI). For AcrBper, only the superimposed drugs are shown. Residues which are positively affecting the susceptibilities for LFX after substitution with Ala are indicated as green sticks.

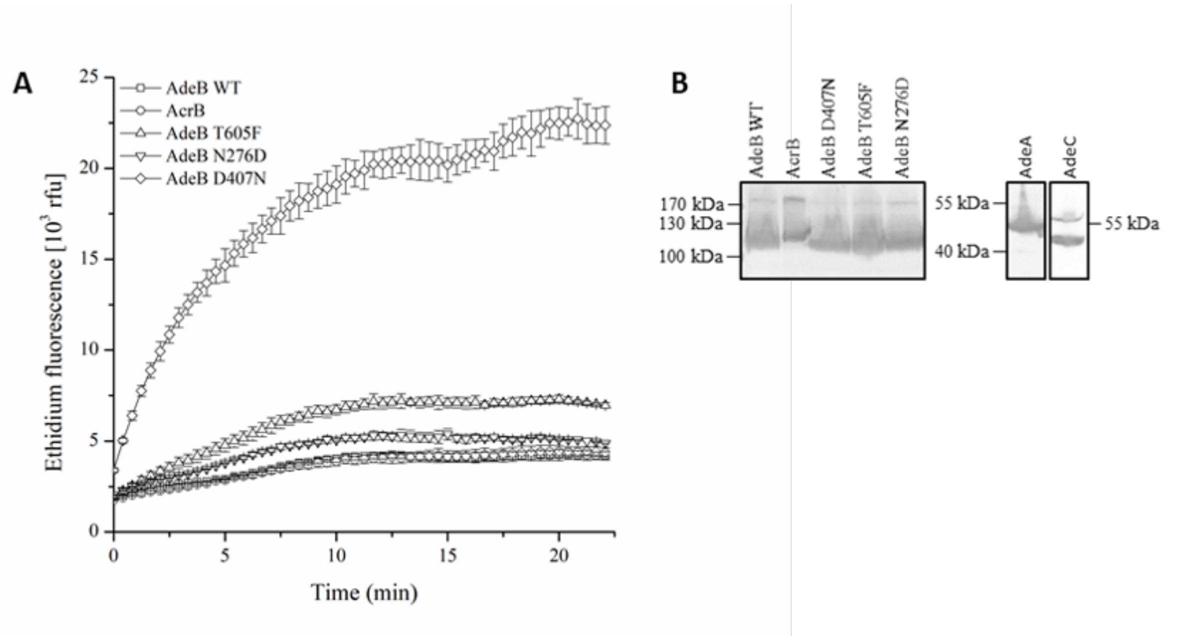

Supplementary Figure 15. **Effect of DBP mutations on the extrusion of ethidium from cells. A** Time-dependent accumulation of ethidium in *E. coli* BW25113 *ΔacrB ΔacrD ΔmdtBC* pRSFD_*adeAC* expressing *acrB*, *adeB* WT, D407N, T605F and N276D visualized by increase of fluorescence intensity. AcrB and AdeB WT mediate efficient efflux of ethidium from the cells, while the inactive variant AdeB D407N cannot abolish the cellular accumulation of the drug. AdeB T605F and, to a lower extent, AdeB N276D showed a reduced viability in the presence of ethidium. This can be confirmed by the increased ethidium accumulation in cells producing these mutants. Experiments were performed in triplicate (n=3, biological triplicates, data available in a Source Data file, one biological experiment is shown here); error bars represent the standard deviation of two technical replicate measurements. Rfu: relative fluorescence units **B** Validation of protein expression by Western Blot analysis. AcrB, AdeB WT and mutants were detected via His-tag, AdeA and AdeC via Myc- und Strep-tag. Uncropped images of the blots are available in a Source Data file.

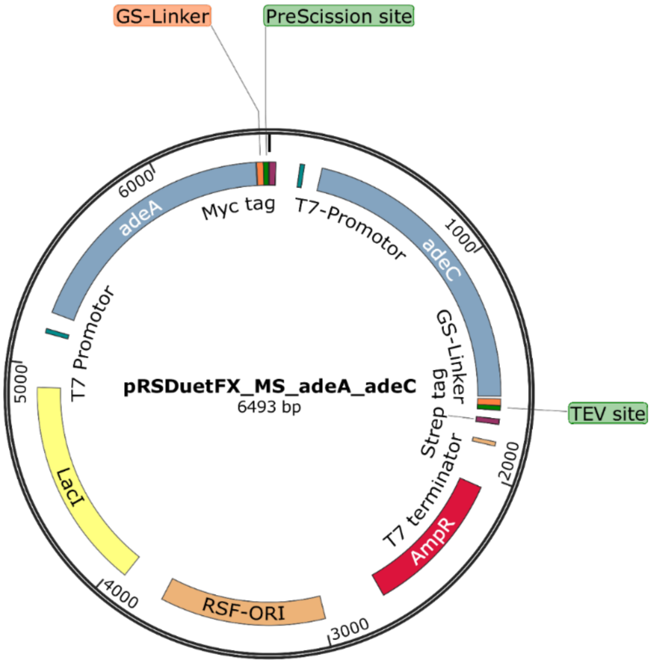

Supplementary Figure 16. **Schematic overview of pRSFDuetFX_MS_*adeA_adeC* for the heterologous co-expression of *adeAC*.** The vector is based on pRSFDuet-1 (Novagen) and was modified in several steps. It contains a FX-cloning compatible multiple cloning site (MCS) coupled to a sequence encoding a PreScission protease cleavable Myc-tag. A second, pET24a-derived MCS was incorporated and modified to contain restriction sites for KpnI, NdeI and PacI. Furthermore, a tobacca etch virus (TEV) protease-cleavable Strep tag-encoding sequence was fused to the 5' end of the MCS. The gene *adeA* was cloned in the FX-cloning compatible MCS, so that the resulting gene product will be fused to a Myc-tag. The *adeC* gene was inserted into the pET24a-derived MCS using KpnI and PacI. The resulting gene product will be fused to a Strep-tag. Additionally, the kanamycin resistance cassette was exchanged with an ampicillin resistance gene.

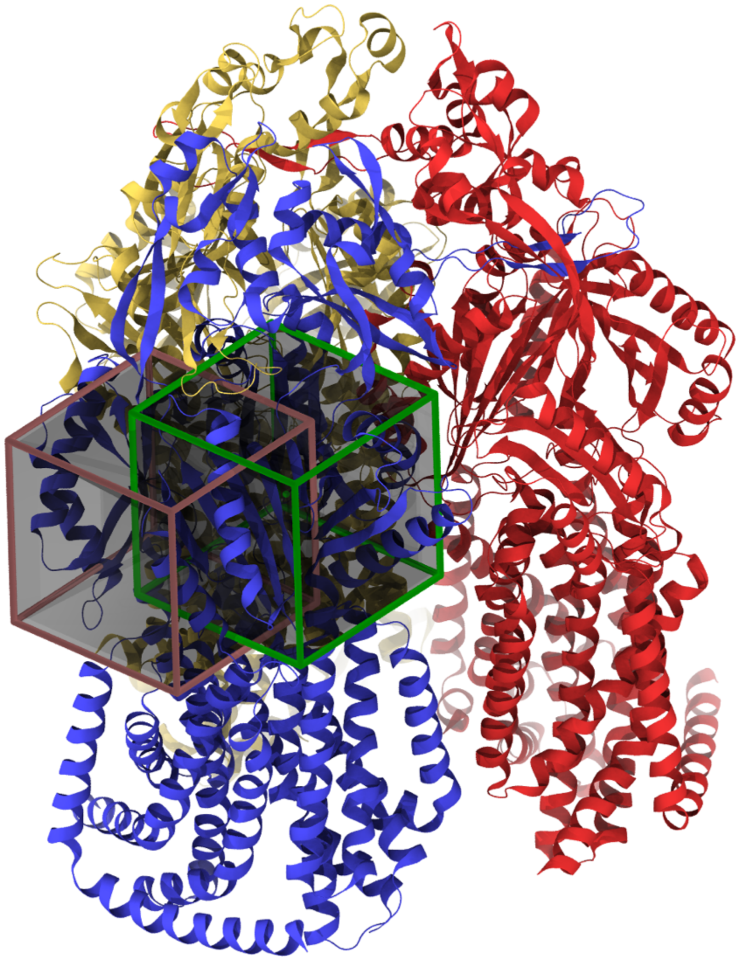

Supplementary Figure 17. **Docking volumes.** Visual representation of the two docking volumes employed in docking calculations with both Autodock VINA and GNINA. The centres of the two rectangular boxes, both of dimensions 30 x 30 x 30 Å^3, have been taken from the centers of mass of ETH molecules bound in the cryo-EM structures of AdeB (PDB_IDs: 7KGI and 7KGG) and while reported on the L monomer here, they are centered on the L (dark red box) and T (dark green) monomers, respectively. L, T, and O monomers are colored blue, yellow and red, respectively.

Supplementary Table 1: **Statistics of cryo-EM data collection and processing from two merged datasets.**

Cryo-EM data collection/processing	
Electron microscope	Titan Krios with K2 detector
Voltage [kV]	300
Magnification [x]	130,000
Pixel size [Å]	1.05
Defocus range [µm]	-1.0 to -3.5 / -1.5 to 4.0
Energy filter width [eV]	20
Exposure time [s]	10.6 / 8.16
Dose rate [(e^-/Å^2)/s]	5.65 / 7.45
Number of frames per image	48 / 48
Total dose	60 / 60.8
No. of micrographs	990 /1007
Initial particle number	381,631
FSC threshold	0.143
AdeB OOO	
Final particle number	132,346
Symmetry	C3
Resolution [Å]	3.54
AdeB T	
Final particle number	35,170
Symmetry	C1
Resolution [Å]	3.95
AdeB L*OO	
Final particle number	34,890
Symmetry	C1
Resolution [Å]	3.84

Supplementary Table 2. **RMSDs (in Å) of the AdeB O and L* conformations (C_α-atoms) compared to published structures of RND transporters.** C_α RMSDs were calculated with SUPERPOSE (https://www.ebi.ac.uk/msd-srv/ssm/cgi-bin/ssmserver, the numbers marked with an asterisk (*) are calculated with Pymol, www.pymol.org)

Protein	Conformation	PDB	Reference	**RMSD AdeB O**	**RMSD AdeB L***
AcrB	L	4DX5	10	1.1	0.89
AcrB	T	4DX5	10	1.25	0.68
AcrB	O	4DX5	10	0.65	1.27
AdeB	O	6OWS	29	1.14	2.69
MexB	L	6IIA	31	1.71	1.93
MexB	T	6IIA	31	1.31	0.74
MexB	O	6IIA	31	0.68	1.28
CmeB	O	5LQ3	43	0.95	1.41
CmeB	Resting	5LQ3	43	1.6	1.1
MtrD	L	4MT1	32	1.37	1.45
CusA	apo (O)	3K07	33	0.46	1.19
CusA	Cu(I) (L)	3K0I	33	3.05*	2.60*
AdeB-I	O	7KGD	30	0.91	2.59
AdeB-Et-I	O	7KGG	30	1.07	2.79
	T	7KGG		2.79	1.55
AdeB-Et-II	Resting	7KGH	30	1.97	2.84
	T			2.85	1.76
	O			1.35	2.96
AdeB-Et-III	L	7KGI	30	2.55	2.13
	T			2.83	1.81
	O			1.39	3.01

Supplementary Table 3. **Statistics of AdeB cryo-EM structures in OOO and L*OO conformations.**

Structure statistics	AdeB OOO	AdeB L*OO
Overall resolution (Å)	3.54	3.84
RMSD		
Bond length (Å)	0.006	0.005
Bond angles (°)	1.132	1.139
Validation		
MolProbity score	1.51	1.62
Clash score	4.10	5.63
Rotamers outliers (%)	0.75	0.79
Cß outliers (%)	0.00	0.00
Ramachandran plot (%)		
Allowed	4.55	4.56
Favored	95.45	95.44
Disallowed	0.00	0.00
CC (mask)	0.84	0.80
CC (box)	0.76	0.74
CC (volume)	0.81	0.78

Supplementary Table 4. **Redocking of ETH into AdeB.** Top five docking poses obtained after docking ETH in the cryo-EM structure of AdeB (PDB_ID: 7KGI), using both Autodock VINA and GNINA packages. ETH was considered flexible during docking, while the receptor was assumed rigid. The top (bottom) tables refer to the ligand bound in the AP (DBP) of the L* (or T) monomer. Docking affinities and root-mean-squared-displacements (RMSD) are expressed in kcal/mol and Å, respectively. Predicted binding modes with RMDS < 2.5 Å are highlighted in green. In the case of the GNINA software, binding modes are ordered according to both binding affinity and convolution neural network (CNN) score.

AUTODOCK VINA			GNINA AFFINITY			GNINA SCORE		
mode	affinity	RMSD	mode	affinity	RMSD	mode	CNN score	RMSD
1	-8.4	6.75	1	-8.73	2.31	1	0.9588	4.85
2	-8.3	2.27	2	-8.69	2.30	2	0.8918	2.30
3	-8.3	2.27	3	-8.51	5.38	3	0.8904	5.23
4	-8.2	7.93	4	-8.45	1.62	4	0.8897	2.31
5	-8.1	5.38	5	-8.45	5.23	5	0.8803	4.00

AUTODOCK VINA			GNINA AFFINITY			GNINA SCORE		
mode	affinity	RMSD	mode	affinity	RMSD	mode	CNN score	RMSD
1	-8.4	0.72	1	-8.86	0.72	1	0.8810	0.72
2	-8.1	5.08	2	-8.84	0.87	2	0.8750	0.87
3	-7.8	2.06	3	-8.51	5.08	3	0.6340	5.08
4	-7.8	5.70	4	-8.25	2.04	4	0.4985	13.28
5	-7.8	5.72	5	-8.03	5.89	5	0.4934	2.04

Supplementary Table 5. **Crystallographic data collection and refinement statistics.** Values for the highest-resolution shell are shown in parentheses.

	Doxycycline, DXT	Fusidic acid, FUA	Levofloxacin, LFX
pdb entry	7B8R	7B8S	7B8T
**Data collection**			
Beamline	DESY, P13	DESY	Soleil, PXI
Wavelength (Å)	0.9762	0.9762	0.9786
Resolution range (Å)	48.8 - 2.1 (2.175 - 2.1)	49.83 - 2.3 (2.382 - 2.3)	49.75 - 2.7 (2.797 - 2.7)
Space group	P 21 21 21	P 21 21 21	P 21 21 21
Unit cell a, b, c (Å) α, β, γ (°)	108.515 145.422 174.173 90 90 90	109.709 145.237 175.405 90 90 90	108.652 145.488 175.156 90 90 90
Total reflections	2171301 (223406)	1119560 (114224)	501524 (51221)
Unique reflections	158834 (15593)	124264 (12209)	75440 (7570)
Multiplicity	13.7 (14.3)	9.0 (9.4)	6.6 (6.8)
Completeness (%)	98.81 (98.01)	99.65 (99.20)	97.93 (99.53)
Mean I/sigma(I)	14.87 (1.46)	12.67 (1.61)	9.17 (0.91)
Wilson B-factor	41.76	37.19	60.84
R-merge	0.1238 (2.08)	0.1552 (1.387)	0.192 (2.235)
R-meas	0.1287 (2.156)	0.1644 (1.466)	0.2086 (2.422)
R-pim	0.03475 (0.5647)	0.05321 (0.4697)	0.08072 (0.9251)
CC1/2	0.999 (0.65)	0.997 (0.62)	0.997 (0.502)
CC*	1 (0.887)	0.999 (0.875)	0.999 (0.818)
**Refinement**			
Reflections used in refinement	158777 (15588)	124243 (12209)	75271 (7550)
Reflections used for R-free	7810 (744)	6126 (619)	3744 (390)
R-work	0.2033 (0.2943)	0.1999 (0.2872)	0.2198 (0.3731)
R-free	0.2400 (0.3307)	0.2460 (0.3464)	0.2691 (0.4119)
CC(work)	0.959 (0.807)	0.948 (0.776)	0.939 (0.710)
CC(free)	0.948 (0.763)	0.928 (0.677)	0.895 (0.593)
Number of non-hydrogen atoms	17678	17656	16823
macromolecules	16890	16885	16787
ligands	99	37	26
solvent	689	734	10
Protein residues	2220	2219	2204
RMS(bonds)	0.004	0.004	0.004
RMS(angles)	0.96	0.93	0.94
Ramachandran favored (%)	96.95	97.27	96.79
Ramachandran allowed (%)	2.96	2.69	2.98
Ramachandran outliers (%)	0.09	0.05	0.23
Rotamer outliers (%)	0.00	0.06	0.23
Clashscore	2.28	1.67	3.93
Average B-factor	51.44	39.41	65.29
macromolecules	51.63	39.55	65.27
ligands	59.12 (all)	77.77	82.95
	54.12 (DXT-1)		
	68.40 (DXT-2)		
solvent	45.72	34.18	51.30

Supplementary Table 6. **Analysis of plate dilution assays with *E. coli* AcrB and DBP variants (F136A, F178A, Y327A, F610A, and F628A).** Plate dilution assays were performed with *E. coli* BW25113 *ΔacrB ΔacrD ΔmdtBC* pRSFDuetFX_MS_*adeAC* harboring pET24_*acrB* or mutants. Dilution series of overnight cultures with an OD_{600nm} of 10^{0}, 10^{-1}, 10^{-2}, 10^{-3}, 10^{-4} and 10^{-5} (6 dilution steps) were spotted on Mueller-Hinton agar plates containing 50 µg/ml kanamycin, 50 µg/ml carbenicillin and 20 µM IPTG, with or without (control plate) the tested drug (see Figure S10). Plates were supplemented with the following compounds: 60 µg/ml rhodamine-6G (R6G), 100 µg/ml tetraphenylphosphonium (TPP), 0.01 µg/ml levofloxacin (LFX), and 1 µg/ml chloramphenicol (CAM), All experiments were performed in triplicate. The last dilution steps showing cell growth were documented and averaged. The table indicates the calculated difference to AcrB WT after subtraction of the negative control (D407N). Positive results (green shadings) indicate increased resistance to the drug compared to AdeB WT, negative results (red shadings) indicate decreased resistance. As a comparison, results from Figure 6, with cells harbouring AdeB or AdeB variants are shown.

AcrB variants

Substrate	R6G	TPP	LFX	CAM
Conc [µg/ml]	60	100	0.01	1
AcrB WT	0,00	0,00	0,00	0,00
F136A	-5,33	-0,33	0,00	0,00
F178A	-4,67	-1,22	-4,56	0,00
Y327A	-2,44	-2,22	-2,33	-3,00
F610A	-4,78	-5,11	-5,33	-4,33
F628A	-4,11	-3,22	-2,00	0,00

AdeB variants

Substrate	R6G	TPP	LFX	CAM
Conc [µg/ml]	60	250	0.01	1
AdeB WT	0,00	0,00	0,00	0,00
F136A	-4,33	-4,00	3,67	2,67
F178A	-4,33	-4,00	0,00	-0,33
Y327A	-4,33	-4,00	4,00	2,00
T605A	-0,67	-1,00	0,00	-1,33
T605F	-4,33	-4,00	3,00	3,33
F623A	-4,33	-4,00	0,00	-1,00

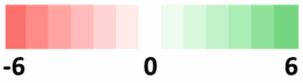

Supplementary Table 7. **List of primers used in this study.**

Insertion of *adeA*, *adeC* into pRSFDuetFX_MS and of *adeb* into p7XC3H		
No.	Primer name	Primer sequence
21	adeA_FX_FW	atatatgctcttctagtgacagtatgcaaaagcatcttttacttc
22	adeA_PX_RV	tatatagctcttcatgctggttgcgccccctc
23	adeB_FX_FW	atatatgctcttctagtatgtcacaatttttattcgtcgtc
24	adeB_FX_RV	tatatagctcttcatgcggatgagattttttcttagaggaaa
25	adeC_KpnI_PacI_FW	atatatggtacctctaaatcggcaatcgtatc
26	adeC_KpnI_PacI_RV	tatatattaattaagacttttgatattcctcctcc

Site-directed mutagenesis of *adeB*		
No.	Primer name	Primer sequence
27	adeB_D407N_FW	aacgatgccattgttgtcg
28	adeB_D407N_RV	gacaataatcccgatggcaag
29	adeB_E89Q_FW	cagattaccgctacgtttaaacc
30	adeB_E89Q_RV	tgctgtaccggaggtatc
31	adeB_G135S_FW	agctttttaatgctggtcgggatt
32	adeB_G135S_RV	ggacgatgaagcttcaacc
33	adeB_Q292K_FW	aaattaagcccgggagctaac
34	adeB_Q292K_RV	aattgcagccgcggtag
35	adeB_W568V_FW	gtgttcatgacttcgttccag
36	adeB_W568V_RV	accttgatcttcctctgg
37	adeB_E151Q_FW	caagttgatttgagtgattatttg
38	adeB_E151Q_RV	ggaatattgattatttggagag
39	adeB_A180S_FW	tctgagaaagctatgcgtatttg
40	adeB_A180S_RV	accgaaagattgaaccttcc
41	adeB_T605F_FW	agtaatttcgccattttggga
42	adeB_T605F_RV	ttttacatcgggattgtctttc
43	adeB_W610F_FW	tttggttttagtggtgcag
44	adeB_W610F_RV	tcccaaaatggcggtattac

45	adeB_N276D_FW	gcatatgactttgccattttgg
46	adeB_N276D_RV	ttgtgaacctatttctacattgg
47	adeB_F277I_FW	gcatataacattgccattttgg
48	adeB_F277I_RV	ttgtgaacctatttctacattgg
49	adeB_E89A_FW	gcgattaccgctacgtttaaacc
50	adeB_E89Q_RV	see Primer No. 30
51	adeB_F136A_FW	ggagcattaatgctggtcggg
52	adeB_G135S_RV	see Primer No. 32
53	adeB_Q176A_FW	gcatctttcggtgcagagaaagc
54	adeB_Q176A/F178A_RV	aaccttccctacaccttcgac
55	adeB_F178A_FW	caatctgccggtgcagagaa
56	adeB_Q176A/F178A_RV	see Primer No. 54
57	adeB_F277A_FW	gcatataacgctgccattttgg
58	adeB_F277I_RV	see Primer No. 48
59	adeB_Q292A_FW	gcattaagcccgggagctaac
60	adeB_Q292K_RV	see Primer No. 34
61	adeB_Y327A_FW	attcctgcagacaccgcg
62	adeB_Y327A_RV	actaaattccatgccttccg
63	adeB_M570A_FW	tggttcgcgacttcgttcc
64	adeB_W568V_RV	see Primer No. 36
65	adeB_T605A_FW	agtaatgccgccattttggga
66	adeB_T605F_RV	see Primer No. 42
67	adeB_F623A_FW	gtagctgtggctgcaacgaca
68	adeB_F623A_RV	attttgtcctgcaccactaa
