# Supplementary material for: Structural and functional analysis of the promiscuous AcrB and AdeB efflux pumps suggests different drug binding mechanisms
Source: Nat Commun. 2021 Nov 25;12:6919. doi: 10.1038/s41467-021-27146-2 (PMC8617272; doi:10.1038/s41467-021-27146-2)
Supplement: Supplementary file 3 — Reporting Summary [file 41467_2021_27146_MOESM3_ESM.pdf]

nature research

Corresponding author(s): Frangakis, Pos

Last updated by author(s): 2021/10/11

Reporting Summary

Nature Research wishes to improve the reproducibility of the work that we publish. This form provides structure for consistency and transparency in reporting. For further information on Nature Research policies, see our Editorial Policies and the Editorial Policy Checklist.

Statistics

For all statistical analyses, confirm that the following items are present in the figure legend, table legend, main text, or Methods section.

n/a	Confirmed	
☐	☒	The exact sample size (*n*) for each experimental group/condition, given as a discrete number and unit of measurement
☐	☒	A statement on whether measurements were taken from distinct samples or whether the same sample was measured repeatedly
☒	☐	The statistical test(s) used AND whether they are one- or two-sided<br>*Only common tests should be described solely by name; describe more complex techniques in the Methods section.*
☒	☐	A description of all covariates tested
☐	☒	A description of any assumptions or corrections, such as tests of normality and adjustment for multiple comparisons
☐	☒	A full description of the statistical parameters including central tendency (e.g. means) or other basic estimates (e.g. regression coefficient) AND variation (e.g. standard deviation) or associated estimates of uncertainty (e.g. confidence intervals)
☒	☐	For null hypothesis testing, the test statistic (e.g. *F*, *t*, *r*) with confidence intervals, effect sizes, degrees of freedom and *P* value noted<br>*Give P values as exact values whenever suitable.*
☒	☐	For Bayesian analysis, information on the choice of priors and Markov chain Monte Carlo settings
☒	☐	For hierarchical and complex designs, identification of the appropriate level for tests and full reporting of outcomes
☒	☐	Estimates of effect sizes (e.g. Cohen's *d*, Pearson's *r*), indicating how they were calculated

Our web collection on statistics for biologists contains articles on many of the points above.

Software and code

Policy information about availability of computer code

Data collection	
Data analysis	X-ray: X-ray datasets were processed using XDS package (Version January 31, 2020 BUILT=20200131) or AIMLESS from the CCP4 suite (version 7.0). REFMAC5 (CCP4 suite 7.0) or phenix.refine from the PHENIX package 1.17.1,, COOT, MolProbity 63. Polder Maps from the PHENIX package 1.17.1,LigPlot+ version 2.2, Coot 0.89, Pymol 2.4.0a0, CCP4i version 7, Phenix 1.17.1, Molprobity, phenix.polder within Phenix package 1.17.1, Refmac5 within CCP4i package version 7.Cryo-EM: UCSF Motioncor2, Gctf, RELION 3.0. Docking: AutoDock VINA 1.1.2 , GNINA 1.0. Molecular dynamics: AMBER18.

For manuscripts utilizing custom algorithms or software that are central to the research but not yet described in published literature, software must be made available to editors and reviewers. We strongly encourage code deposition in a community repository (e.g. GitHub). See the Nature Research guidelines for submitting code & software for further information.

Data

Policy information about availability of data

All manuscripts must include a data availability statement. This statement should provide the following information, where applicable:

- Accession codes, unique identifiers, or web links for publicly available datasets
- A list of figures that have associated raw data
- A description of any restrictions on data availability

Atomic coordinates and structure factors reported in this paper have been deposited in the Protein Data Bank under accession numbers 7B8P [http://doi.org/10.2210/pdb7B8P/pdb] (AdeB-OOO), 7B8Q [http://doi.org/10.2210/pdb7B8Q/pdb] (AdeB-L*OO), 7B8R [http://doi.org/10.2210/pdb7B8R/pdb] (AcrBper/DARPin in complex with Doxycycline), 7B8S [http://doi.org/10.2210/pdb7B8S/pdb] (AcrBper/DARPin in complex with fusidic acid), 7B8T [http://doi.org/10.2210/pdb7B8T/pdb] (AcrBper/DARPin in complex with Levofloxacin).

Atomic coordinates that were used and support the findings of this study are available in the Protein Data Bank under accession numbers 5ENS [http://doi.org/10.2210/pdb5ENS/pdb], 7KGI [http://doi.org/10.2210/pdb7KGI/pdb], 7KGH [http://doi.org/10.2210/pdb7KGH/pdb], 7KGG [http://doi.org/10.2210/pdb7KGG/pdb], 7KGD [http://doi.org/10.2210/pdb7KGG/pdb], 4DX5 [http://doi.org/10.2210/pdb4DX5/pdb], 4DX7 [http://doi.org/10.2210/pdb4DX7/pdb], 5NC5 [http://doi.org/10.2210/pdb5NC5/pdb], 6OWS [http://doi.org/10.2210/pdb6OWS/pdb], 6IIA [http://doi.org/10.2210/pdb6IIA/pdb], 5LQ3 [http://doi.org/10.2210/pdb5LQ3/pdb], 4MT1 [http://doi.org/10.2210/pdb4MT1/pdb], 3K07 [http://doi.org/10.2210/pdb3K07/pdb], 3K0I [http://doi.org/10.2210/pdb3K0I/pdb]. Source data for supplementary Figures 1, 10-13 and 15 are provided with this paper.

Field-specific reporting

Please select the one below that is the best fit for your research. If you are not sure, read the appropriate sections before making your selection.

- [x] Life sciences
- [ ] Behavioural & social sciences
- [ ] Ecological, evolutionary & environmental sciences

For a reference copy of the document with all sections, see nature.com/documents/nr-reporting-summary-flat.pdf

Life sciences study design

All studies must disclose on these points even when the disclosure is negative.

Sample size

X-ray: structure elucidation was done on basis of one dataset per co-crystal, as is usual in the field (in case of molecular replacement phase determination). Completeness of the data was 98-99% and multiplicity between 6.6-13.7 times as indicated in Supplementary Table 5.
Cryo-EM: Micrograph stacks of 48 images were recorded in counting mode using Serial-EM at a magnification of 130,000 x (pixel size of 1.05 Å) with a defocus of -1 to -3.5 (dataset 1) and -1.5 to -4.0 (dataset 2). A total of 1,997 micrograph stacks from both datasets were aligned with UCSF Motioncor2. For structural analysis we attempted to maximize our sample size given the time on the microscope.
The number and identity of the particles that went into each refined map were determined through 3D classification, as described under Methods.
Plate dilution assays: Each experiment (each clone/variant) for bacterial growth on medium agar plates was done 3 times (on different days) These were biological replicates i.e. for each experiment, bacteria were freshly transformed and grown overnight before each dilution experiment. N=3 was considered sufficient, since the results were reproducible.

Data exclusions

No data exclusion.

Replication

Plate dilution assays: Each experiment (each clone/variant) on bacterial growth on medium agar plates was done 3 times on different days (biological replicates). For each experiment, bacteria were freshly transformed and grown overnight before each dilution experiment. Data replication was successful and all experimental results are available in Source Data.
Western Blot analysis: Western blot analysis was repeated three times, except for Suppl. Figure 10b, which was only done once (in the revision stage of the manuscript).

Randomization

In drug agar plate assay, each of the cells from independent AcrB variants are clones or transformants. In principle, colonies were picked randomly from the agar plates after transformation of plasmid into E. coli cells.

Blinding

Not applicable to all the experiments due to the need for rationale design. Negative and positive controls were included in each of the experiments.

Reporting for specific materials, systems and methods

We require information from authors about some types of materials, experimental systems and methods used in many studies. Here, indicate whether each material, system or method listed is relevant to your study. If you are not sure if a list item applies to your research, read the appropriate section before selecting a response.

Materials & experimental systems

n/a	Involved in the study	
[ ]	[x]	Antibodies
[x]	[ ]	Eukaryotic cell lines
[x]	[ ]	Palaeontology and archaeology
[x]	[ ]	Animals and other organisms
[x]	[ ]	Human research participants
[x]	[ ]	Clinical data
[x]	[ ]	Dual use research of concern

Methods

n/a	Involved in the study	
[x]	[ ]	ChIP-seq
[x]	[ ]	Flow cytometry
[x]	[ ]	MRI-based neuroimaging

Antibodies

Antibodies used

1. 6x-His Tag Monoclonal Antibody (4E3D10H2/E3) Thermo Fisher Scientific, Catalog # MA1-135
2. Precision Protein™ StrepTactin-AP Conjugate, Bio-Rad, Catalog #1610382

3. Anti-Myc Tag Antibody, clone 4A6, Sigma-Aldrich, Catalog #05-724
4. anti-AcrB antibody (Custom made antibody by Neosystems, France)
5. anti-rabbit IgG alkaline phosphatase antibody (Product # A3687, Sigma-Aldrich, USA)

Validation

1. https://www.thermofisher.com/antibody/product/6x-His-Tag-Antibody-clone-4E3D10H2-E3-Monoclonal/MA1-135
2. https://www.bio-rad.com/webroot/web/pdf/lsr/literature/MS4110000.pdf
3. https://www.sigmaaldrich.com/catalog/product/mm/05724
4. Anti-AcrB has been validated in our lab with purified AcrB as sample (e.g. Seeger MA, et al. (2008) Engineered disulfide bonds support the functional rotation mechanism of multidrug efflux pump AcrB. Nat Struct Mol Biol 15:199–205).
5. Cibelli et al., 2001 (PMID: 11298794 DOI: 10.1046/j.0953-816x.2001.01510.x), Tam et al., 2021 (PMID: 34188038 DOI: 10.1038/s41467-021-24151-3)
