# Supplementary material for: Structural and functional analysis of the promiscuous AcrB and AdeB efflux pumps suggests different drug binding mechanisms
Source: Nat Commun. 2021 Nov 25;12:6919. doi: 10.1038/s41467-021-27146-2 (PMC8617272; doi:10.1038/s41467-021-27146-2)
Supplement: Supplementary file 4 — Source Data [file 41467_2021_27146_MOESM4_ESM.zip › Source_Data_Suppl_Figs_1_10_11_12_13_15.docx]

**Source Data**

**Structural and functional analysis of the promiscuous AcrB and AdeB efflux pumps suggests different drug binding mechanisms**

**Alina Ornik-Cha^1§^, Julia Wilhelm^1§^, Jessica Kobylka^1^, Hanno Sjuts^1,2^, Attilio V. Vargiu^3^, Giuliano Malloci^3^, Julian Reitz^4,5^, Anja Seybert^4,5^, Achilleas S. Frangakis^4,5*^, Klaas M. Pos^1*^**

^1^Institute of Biochemistry, Goethe-University Frankfurt, Max-von-Laue-Str. 9, D-60438 Frankfurt am Main, Germany.

^2^present address: Biologics Research, Sanofi-Aventis Deutschland GmbH, Frankfurt, Germany

^3^Department of Physics, University of Cagliari, 09042 Monserrato (CA), Italy

^4^Buchmann Institute for Molecular Life Sciences, Goethe-University Frankfurt, Max-von-Laue-Str. 15, D-60438 Frankfurt am Main, Germany.

^5^Institute of Biophysics, Goethe-University Frankfurt, Max-von-Laue-Str. 15, D-60438 Frankfurt am Main, Germany.

^§^Contributed equally

* Correspondence to Klaas M. Pos (pos@em.uni‐[frankfurt.de](mailto:pos@em.uni‐frankfurt.de)) or Achilleas Frangakis (achilleas.frangakis@biophysik.org.)

**Source data of supplementary figure 1**

1. **SEC of AdeB in DDM**

Replicate 1


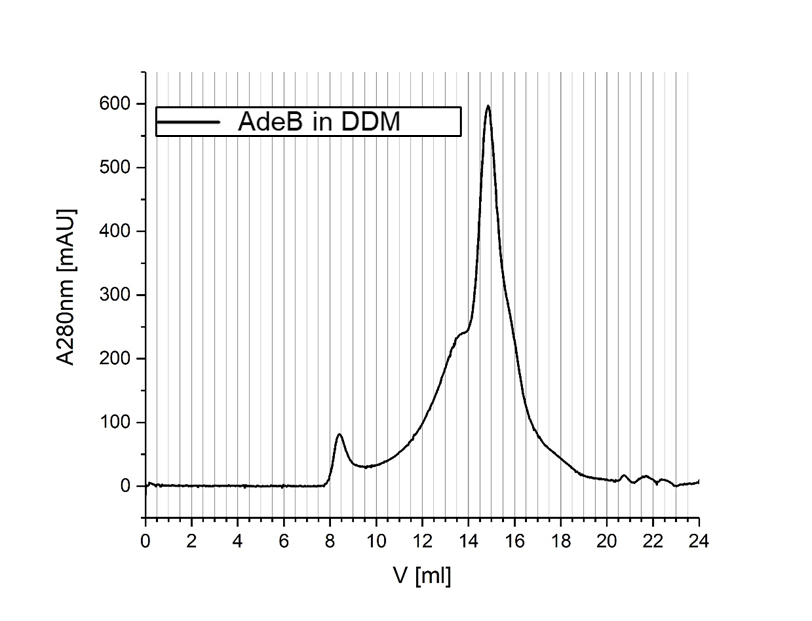


Replicate 2


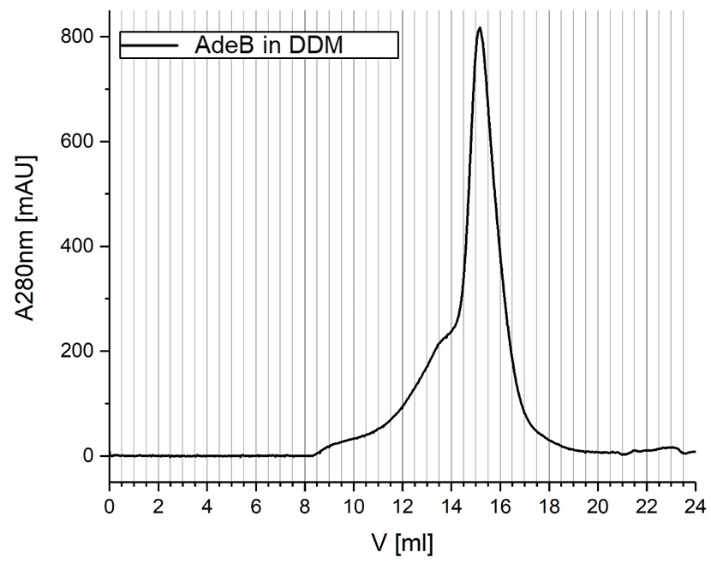


Replicate 3


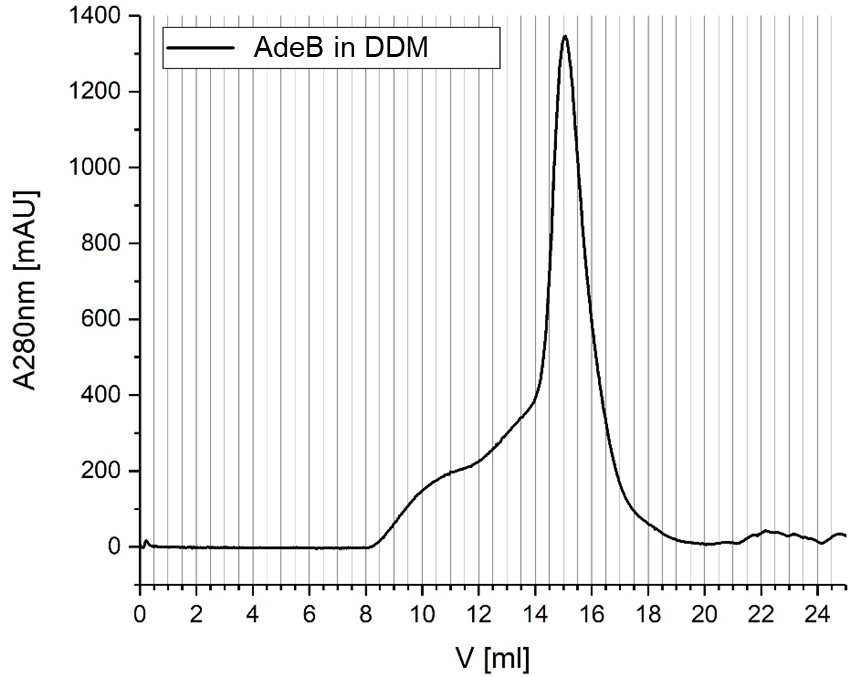


1. **SEC of AdeB in Salipro**

Replicate 1


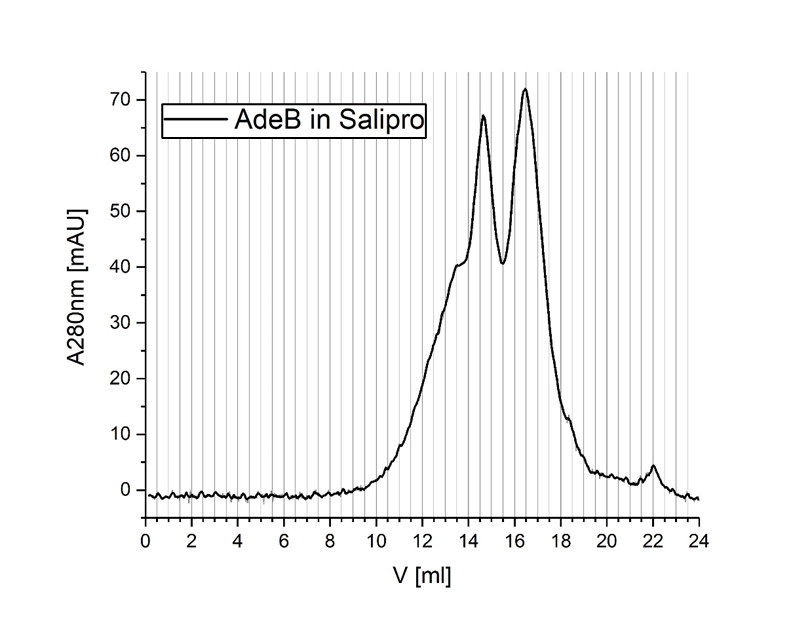


Replicate 2


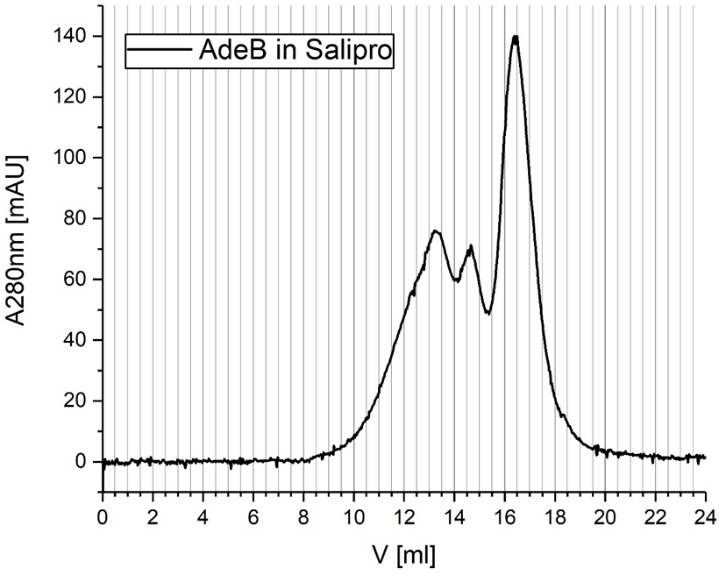


Replicate 3


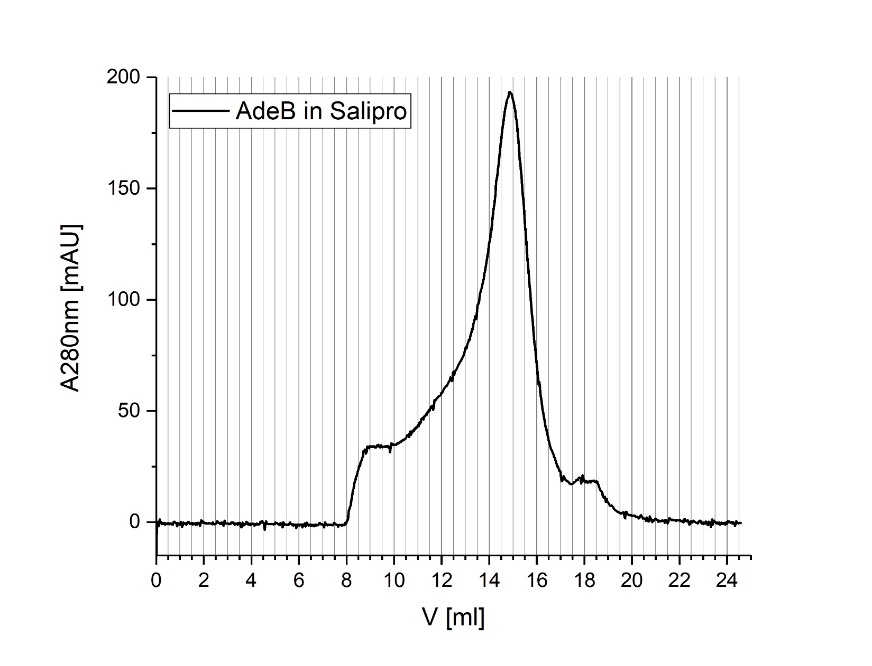


**(c) SDS-PAGE**

Replicate 1

**
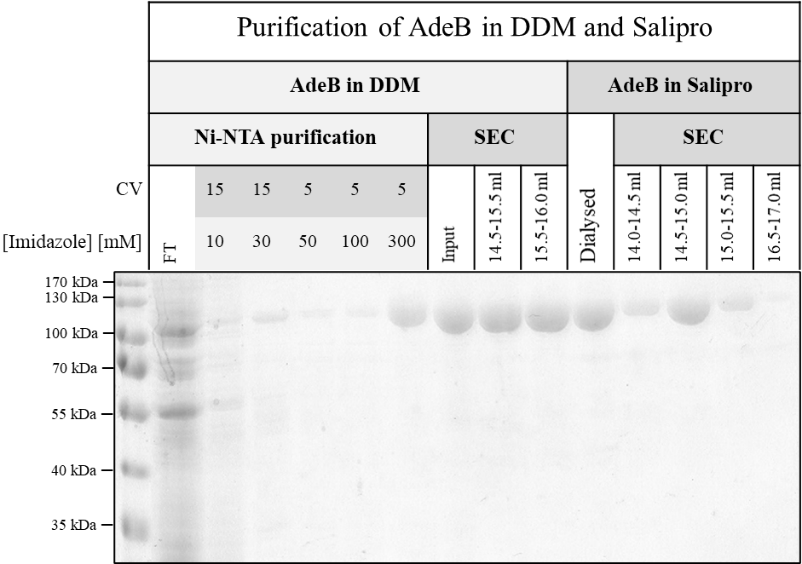
**

Replicate 2


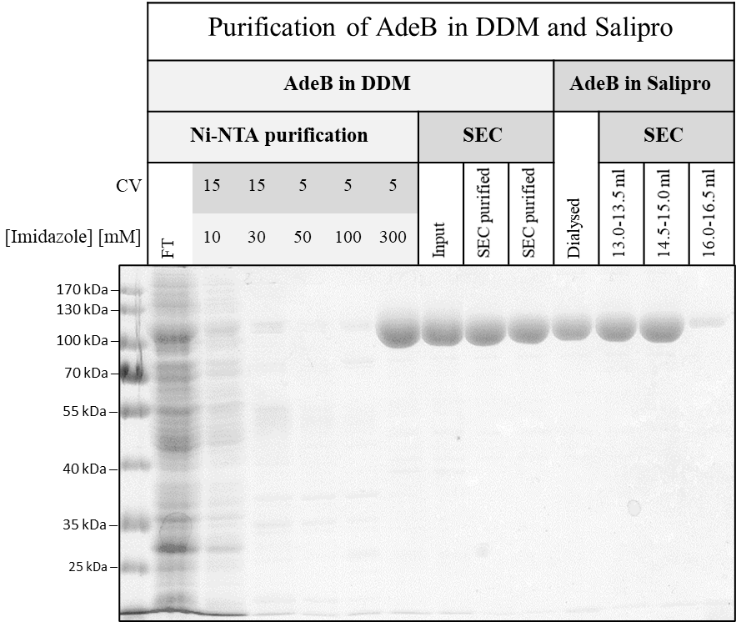


Replicate 3


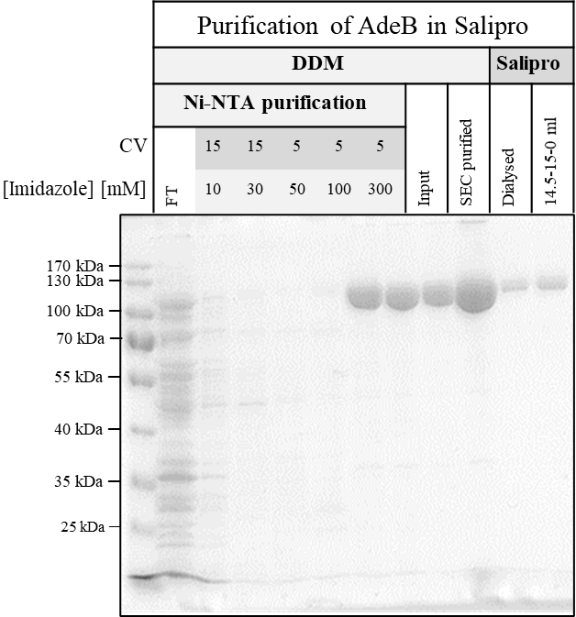


**Source Data of supplementary figure 10**

## Drug agar plate dilution assays


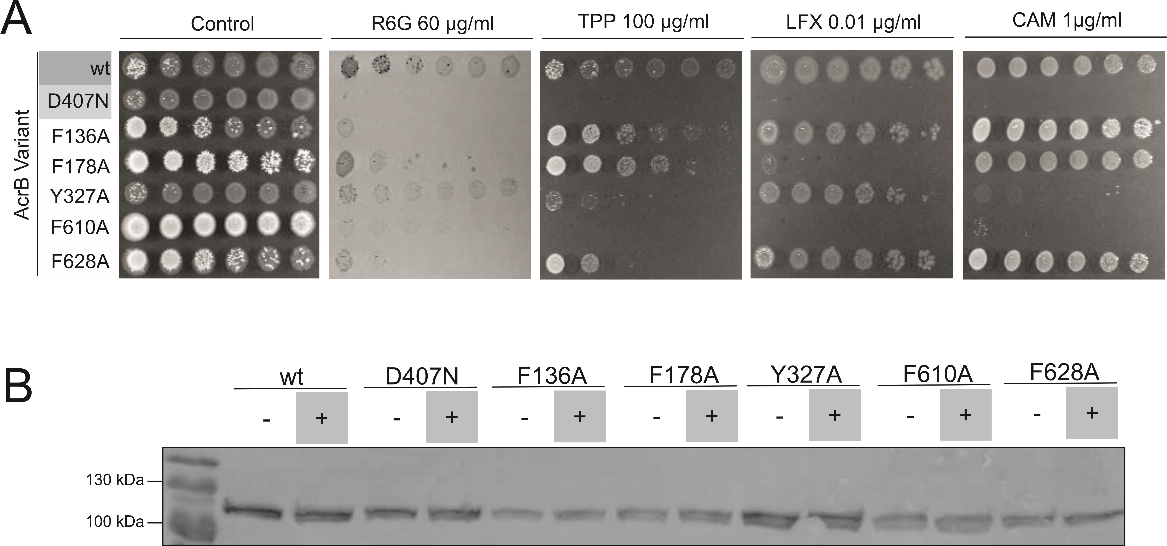


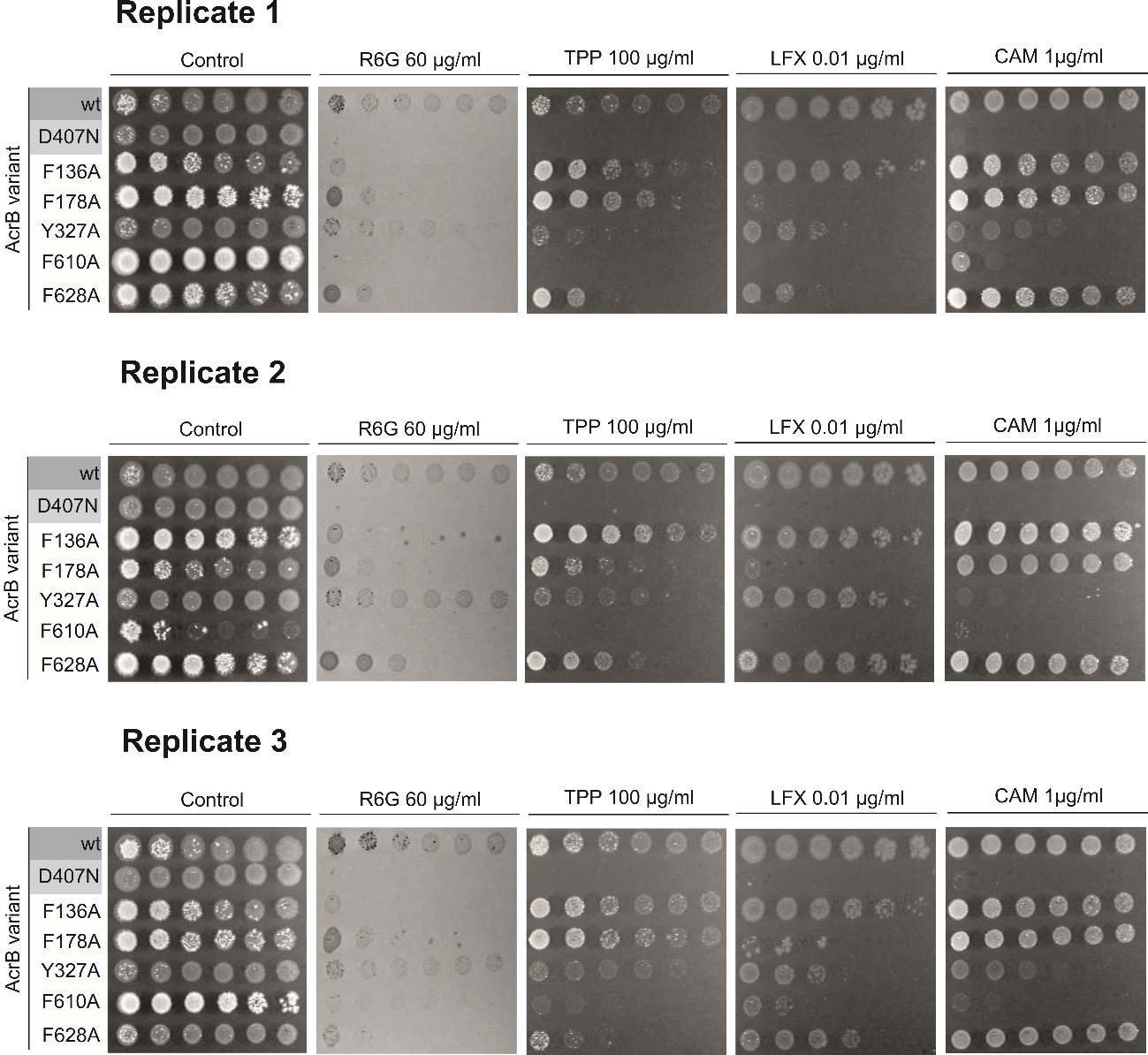


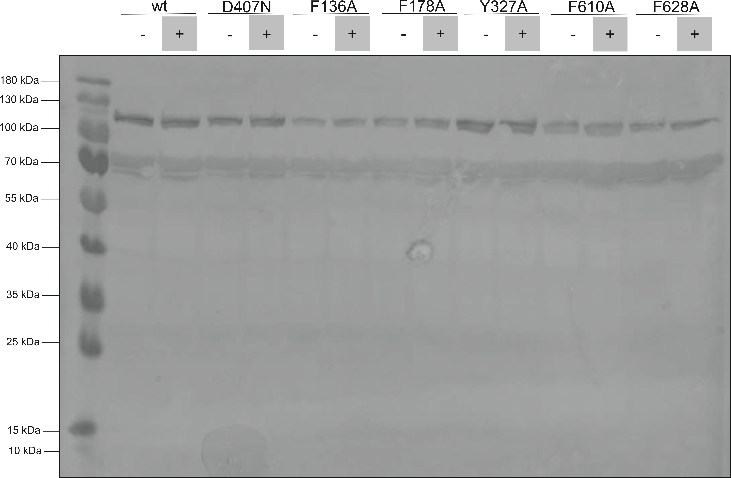


1. **Western Blot with anti-AcrB antibody**

**Source Data of supplementary figure 11**

## Drug agar plate dilution assays

Replicate 1


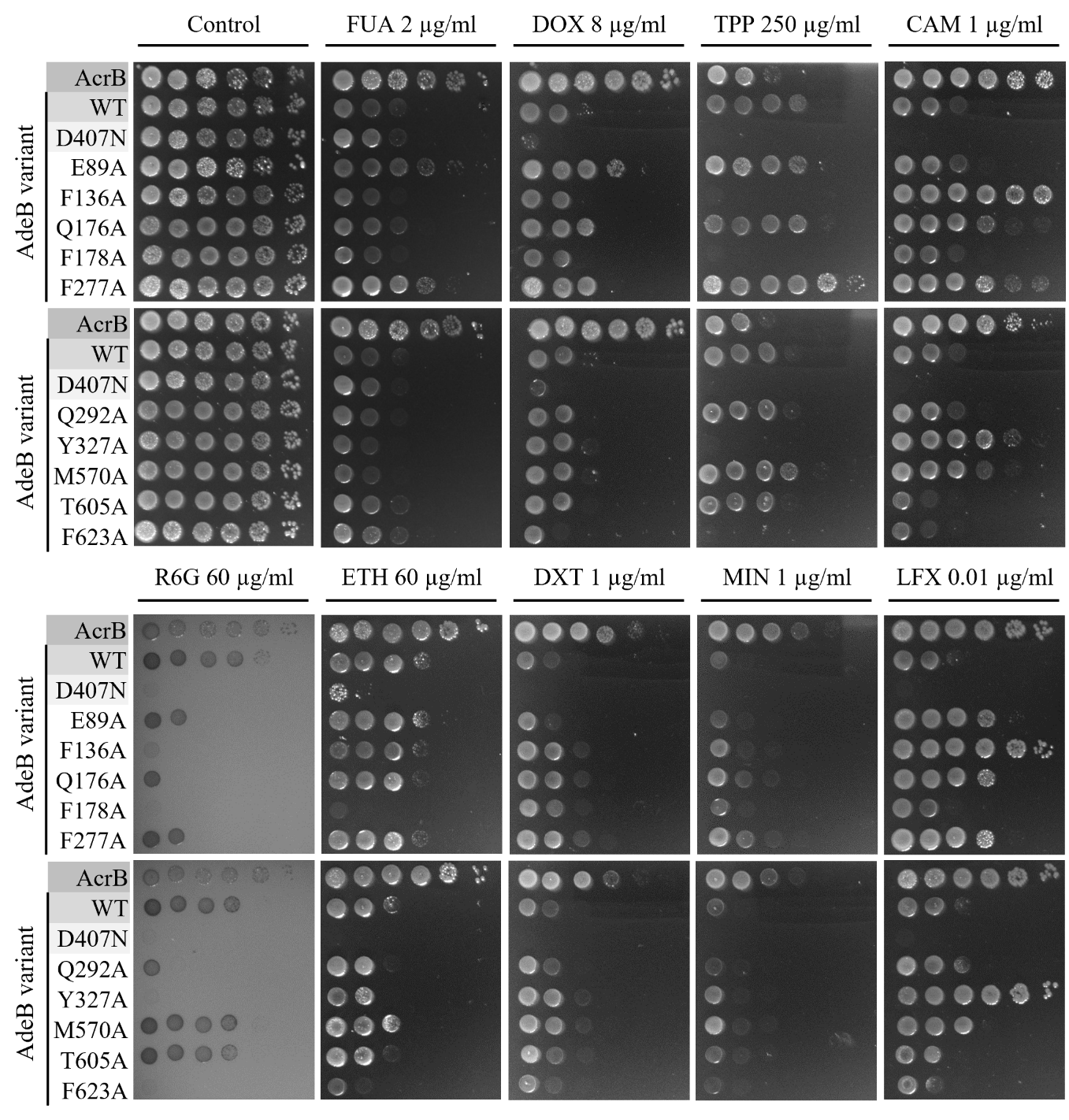


Replicate 2


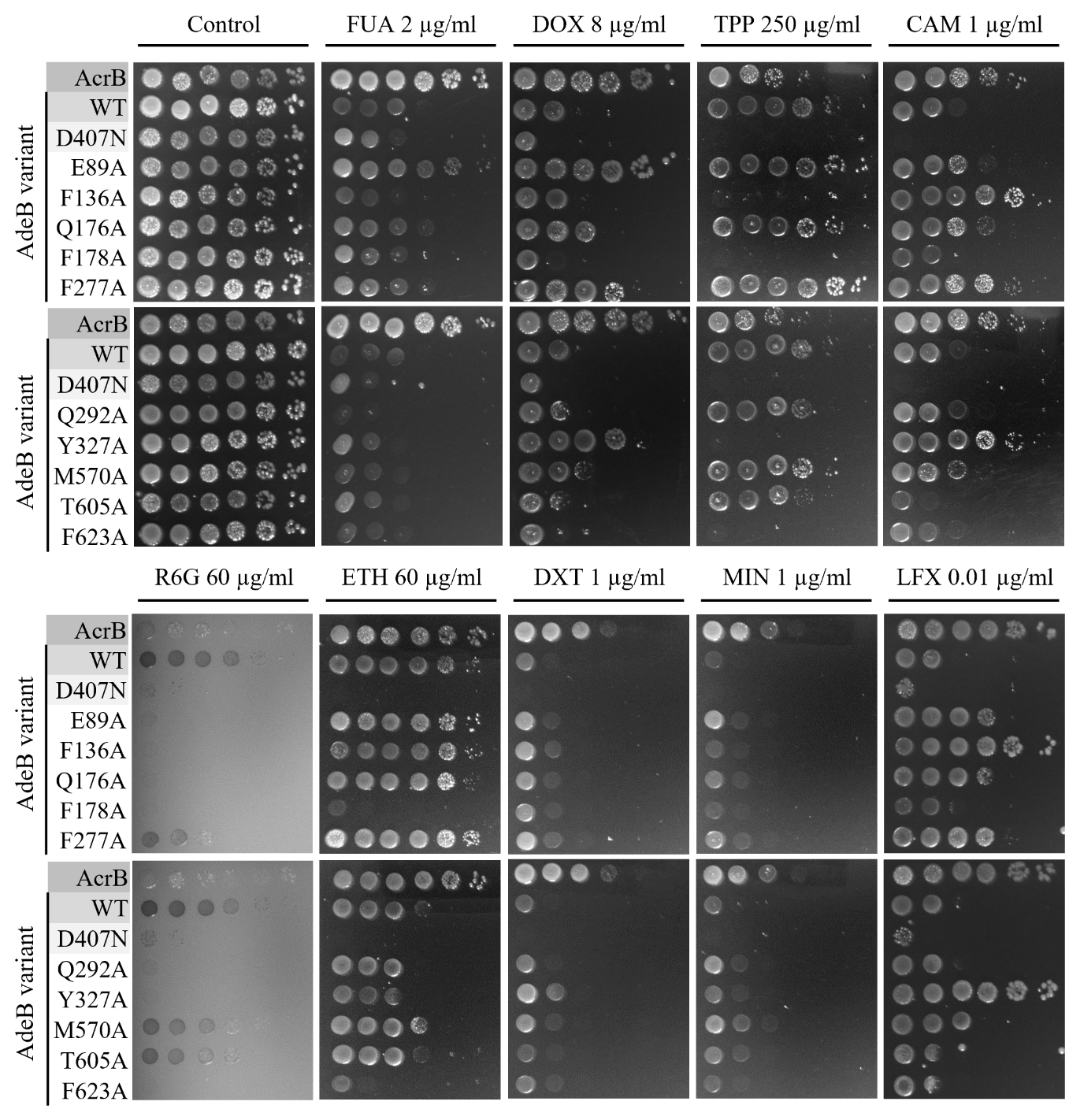


Replicate 3


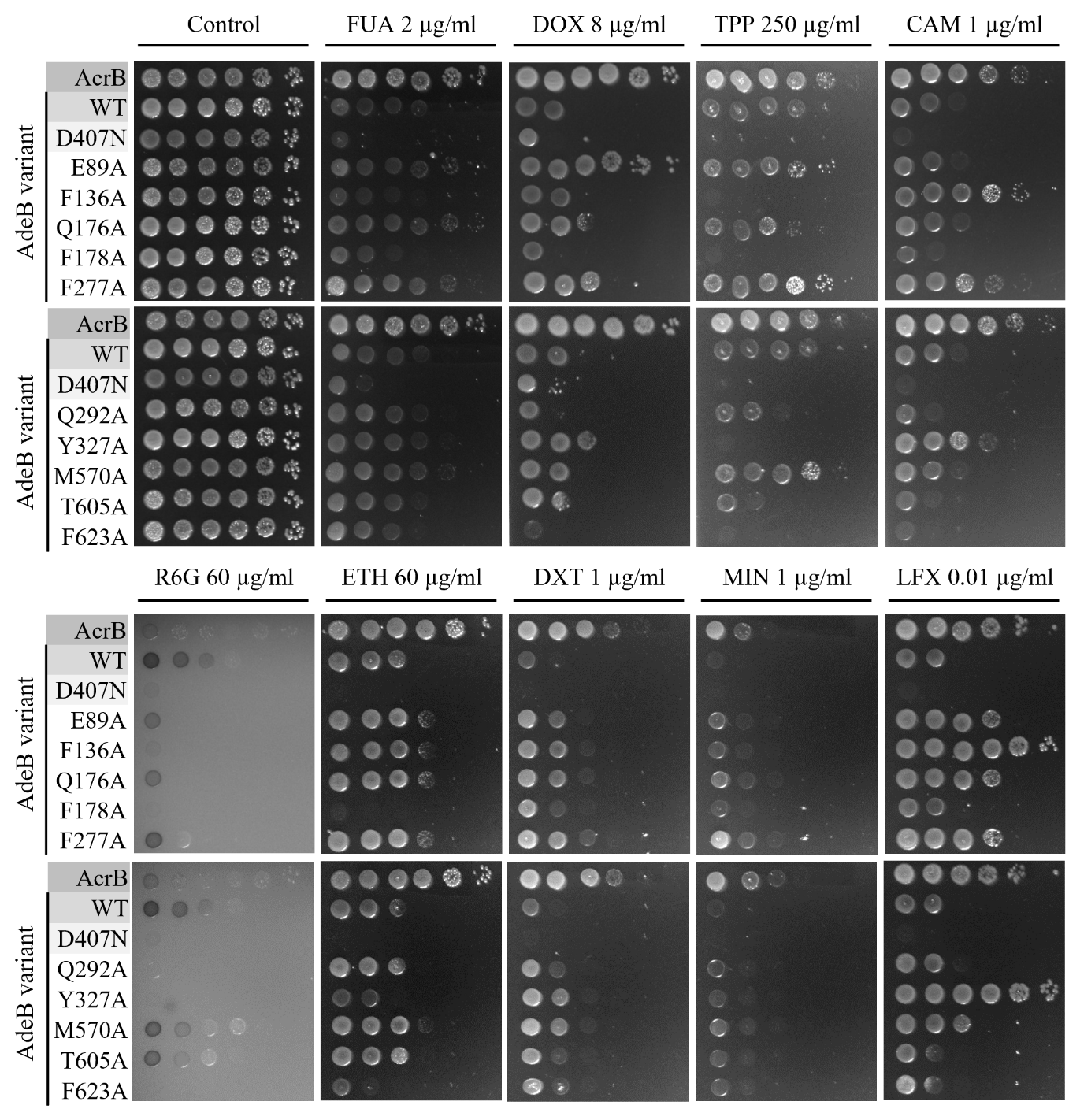


## Western Blot with anti-His-antibody to detect AdeB variants and AcrB

Replicate 1 (whole cells)


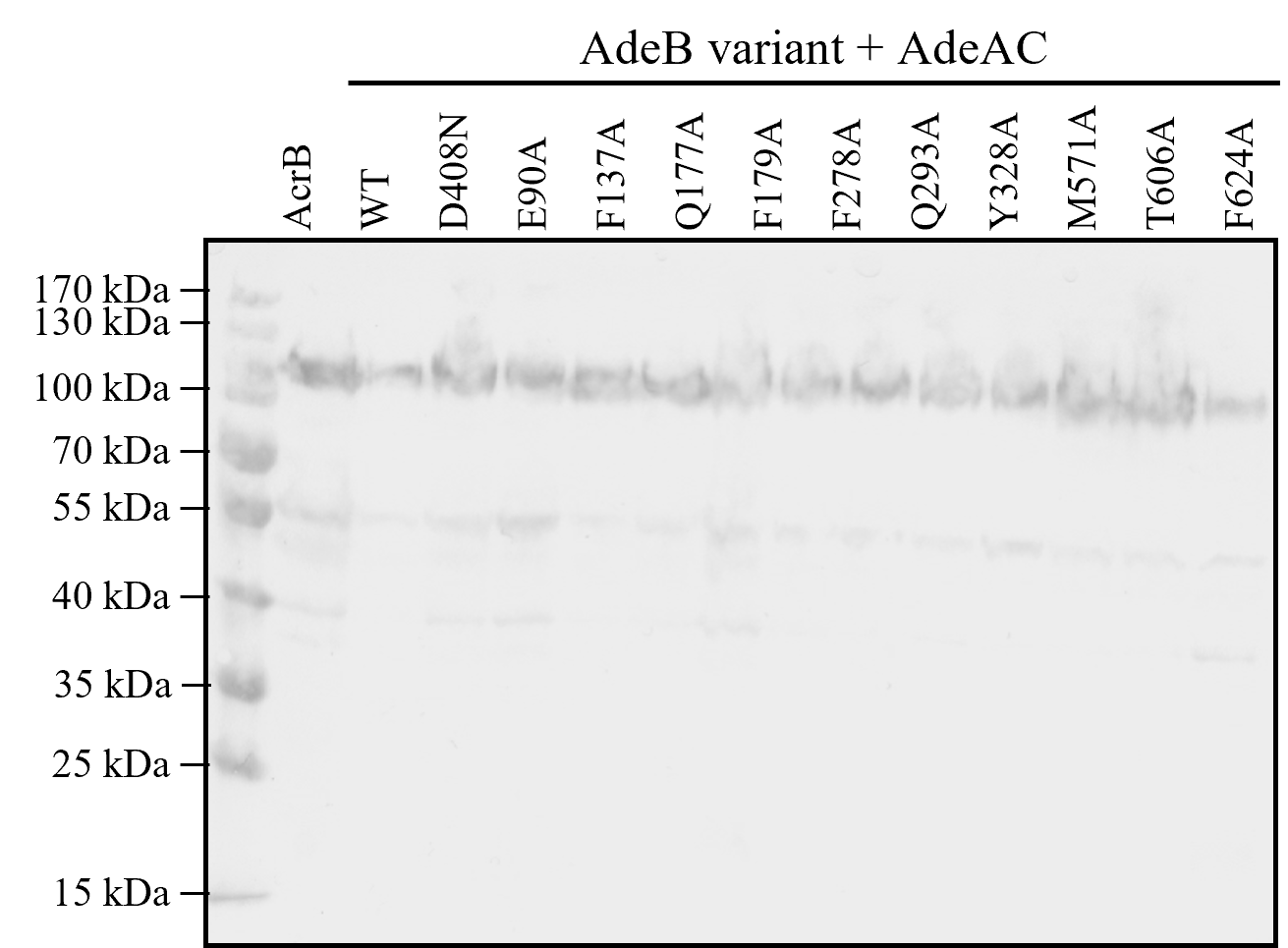


Replicate 2 (whole cells)


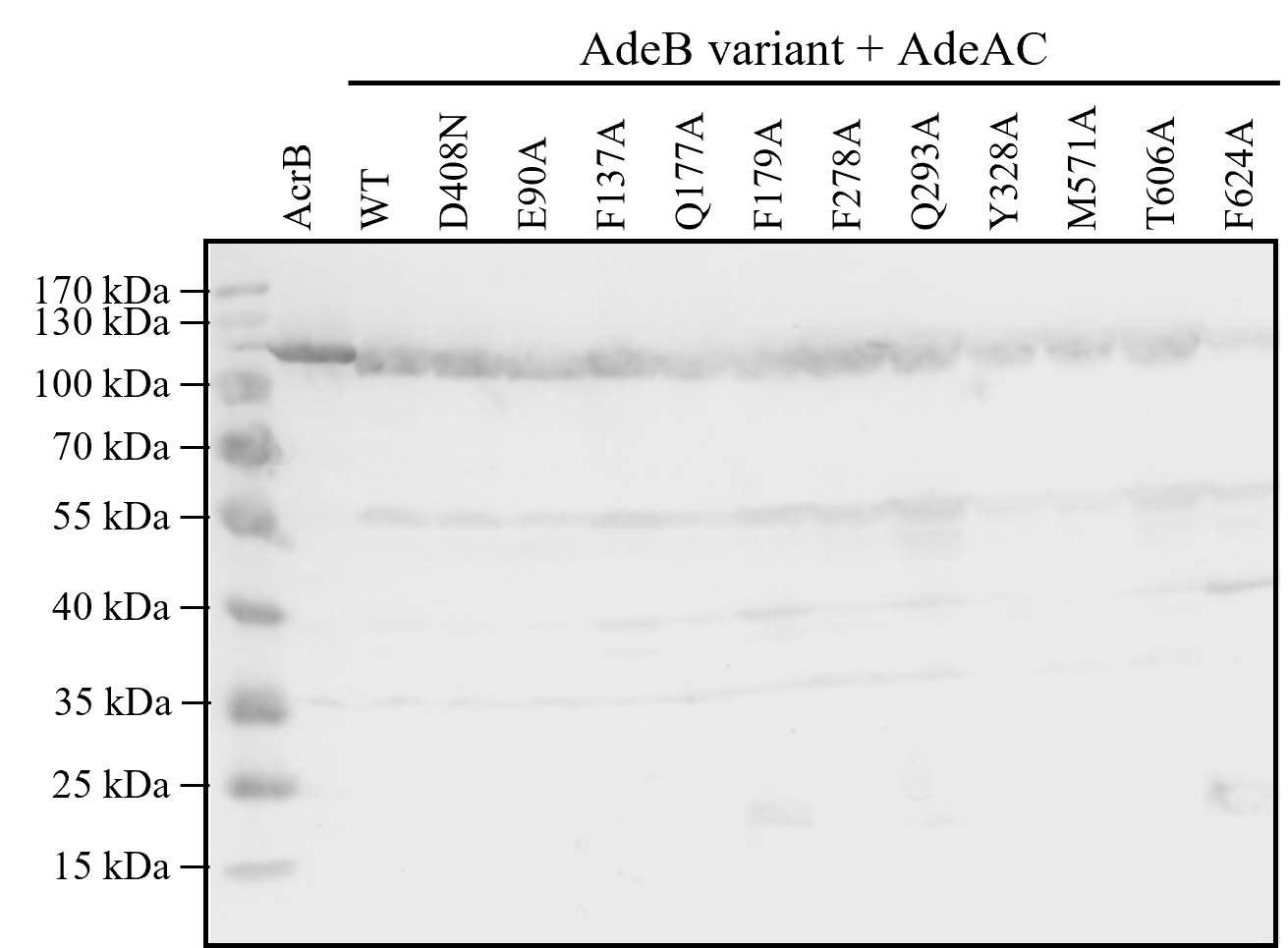


Replicate 3 (solubilized cells)


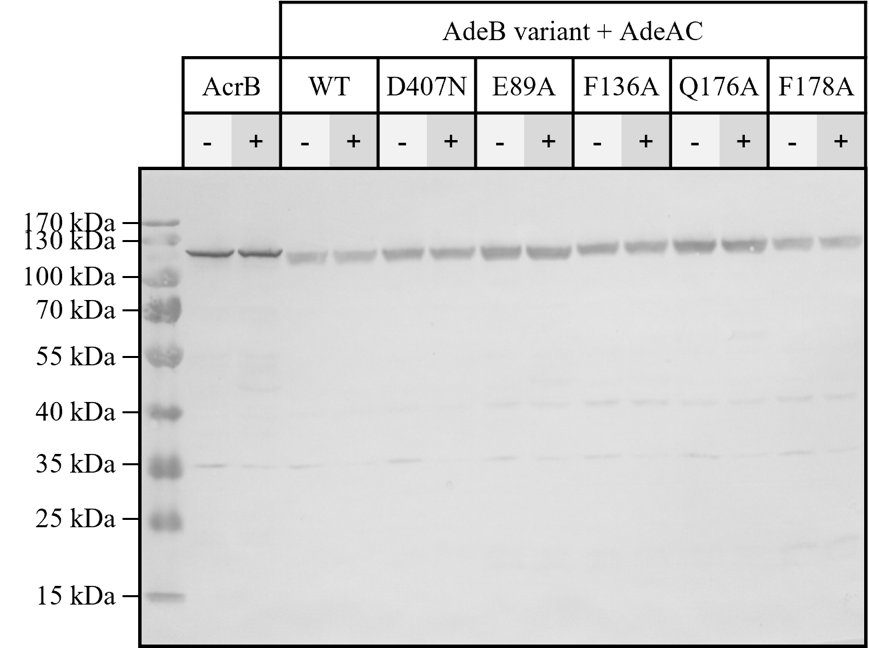


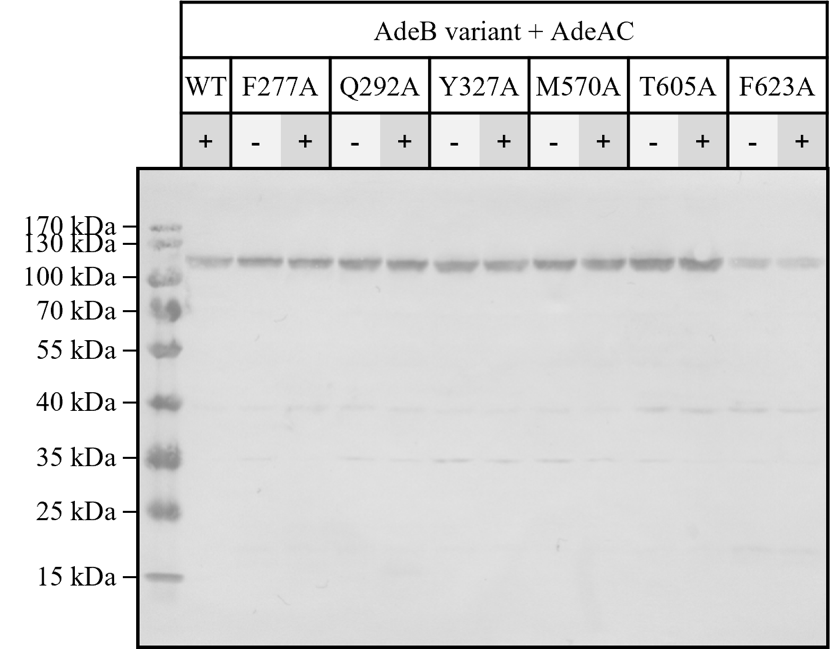


**Source Data of supplementary figure 12**

1. **Drug agar plate dilution assays**

Replicate 1


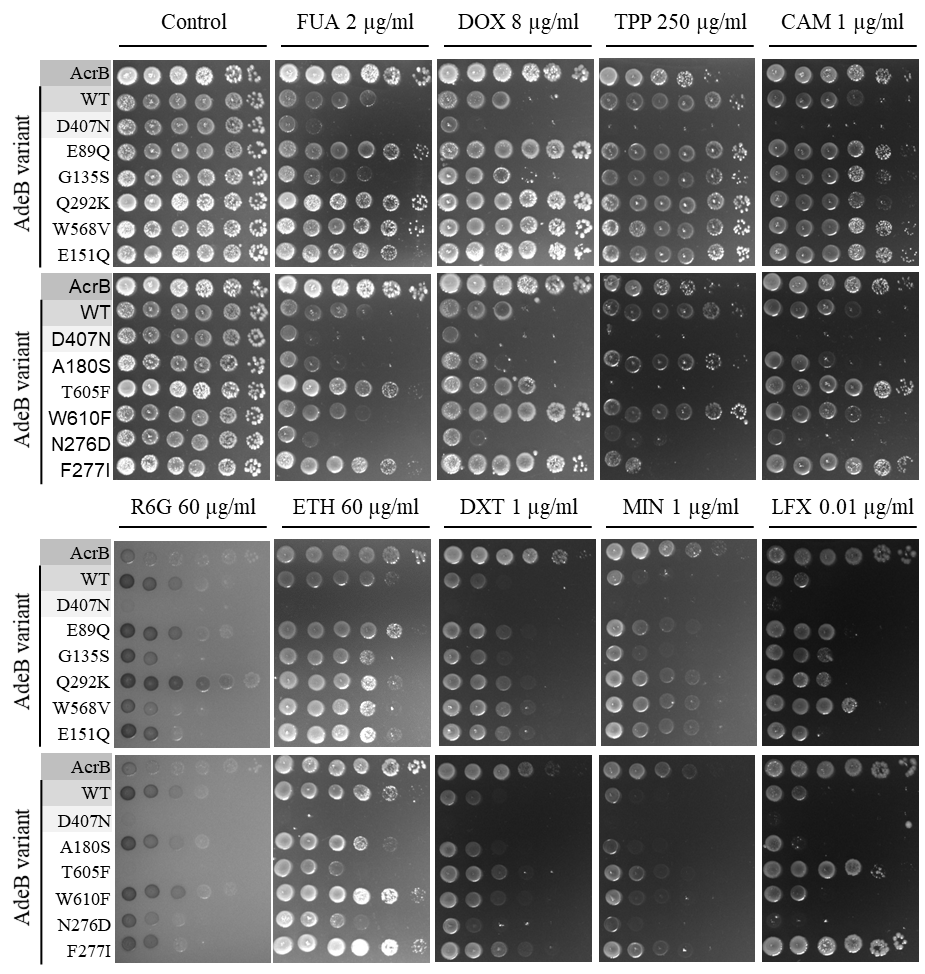


Replicate 2


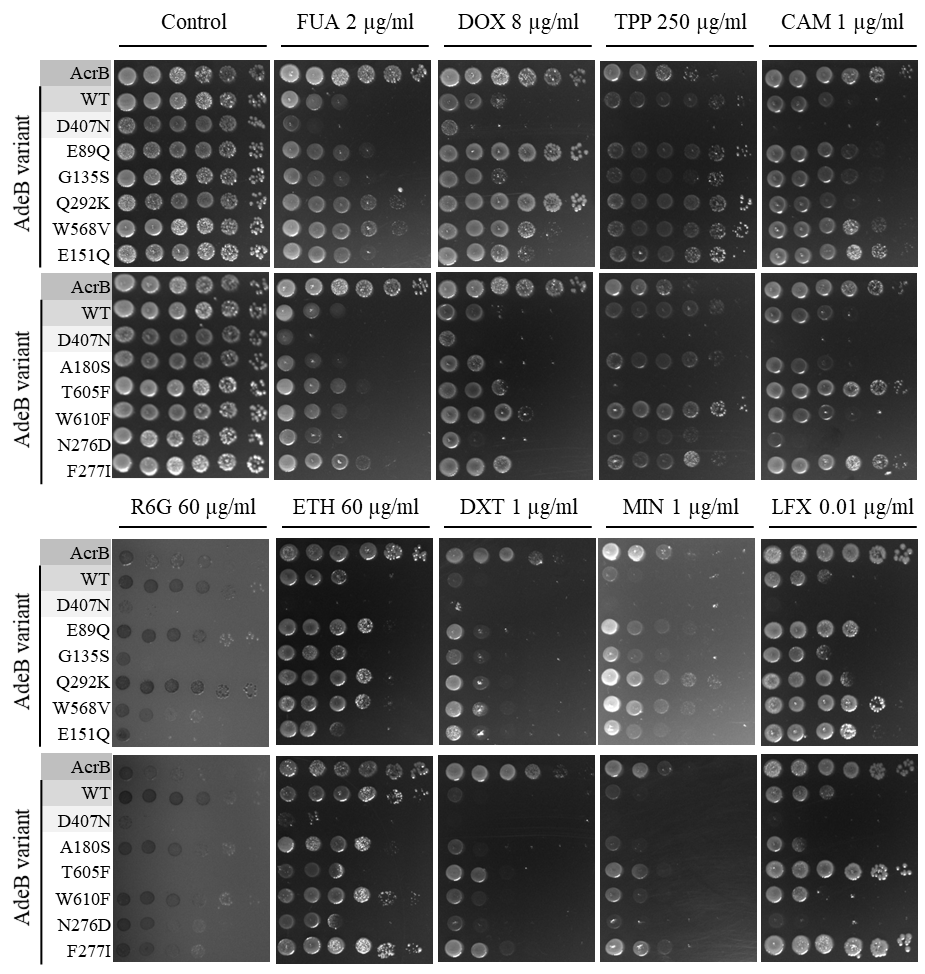


Replicate 3


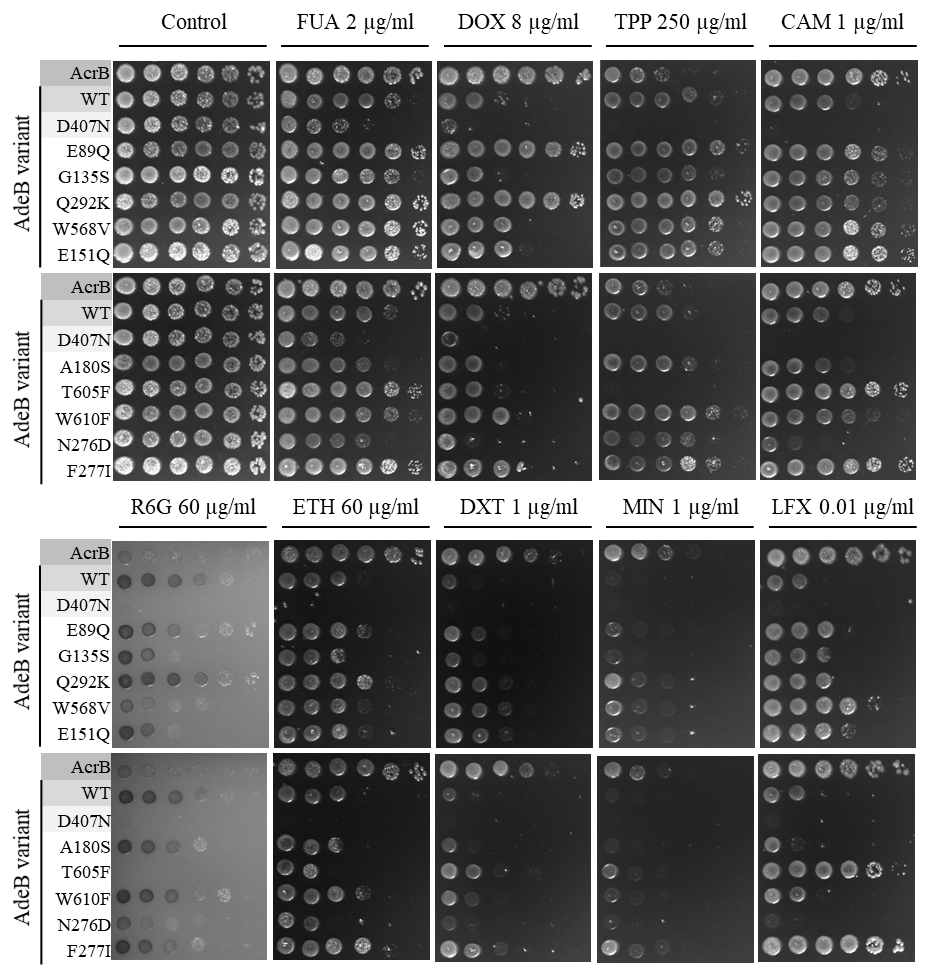


1. **Western Blot with anti-His-antibody to detect AdeB variants and AcrB**

Replicate 1 (whole cells)


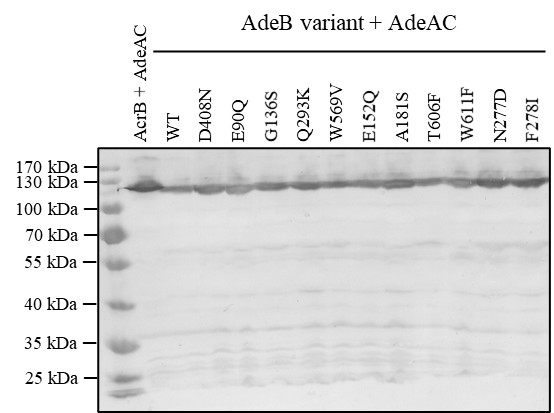


Replicate 2 (whole cells)


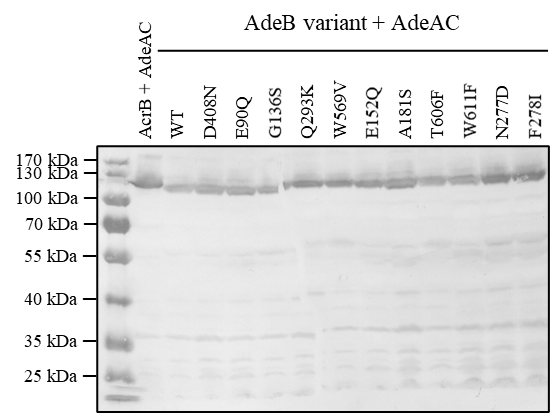


Replicate 3 (solubilized cells)


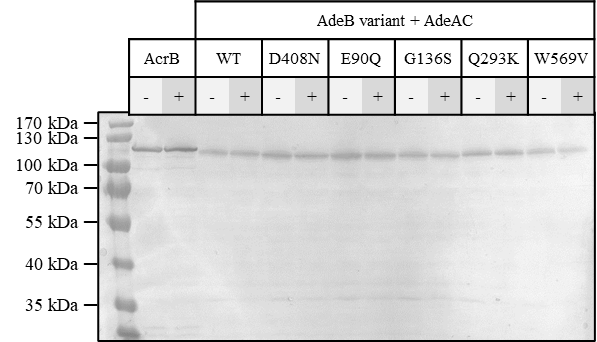


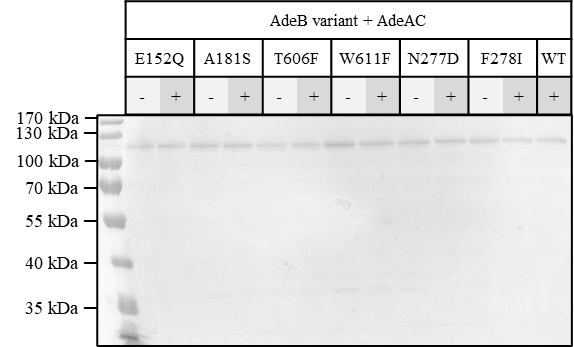


1. **Western Blot with anti-Myc-antibody to detect AdeA (from whole cells)**


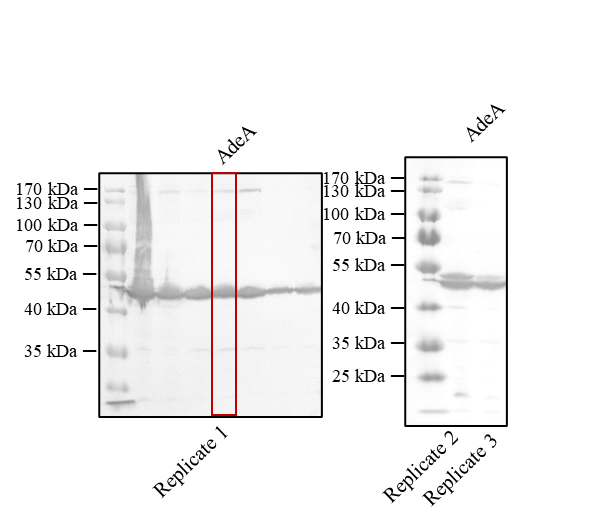


1. **Western Blot with anti-Strep-antibody to detect AdeC (from whole cells)**

**
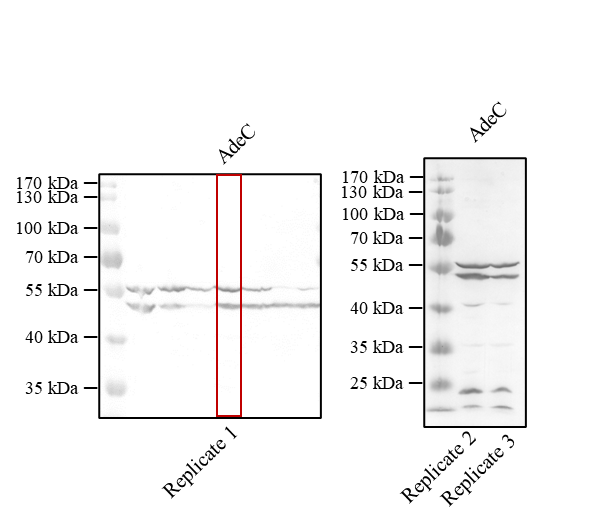
**

**Source data of supplementary figure 13**

## Drug agar plate dilution assays

Replicate 1


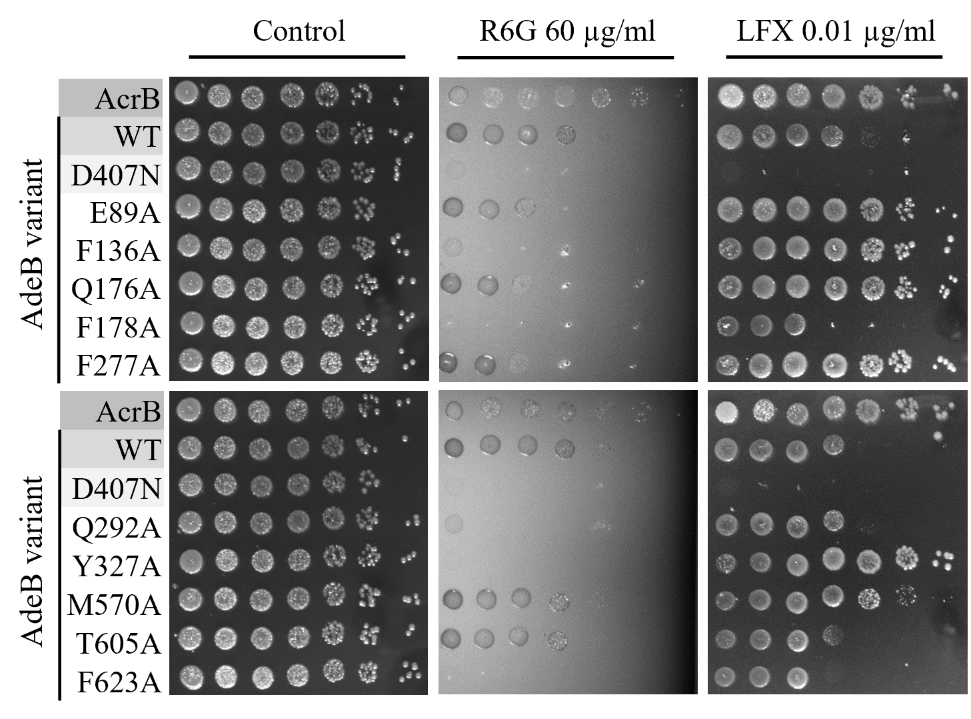


Replicate 2


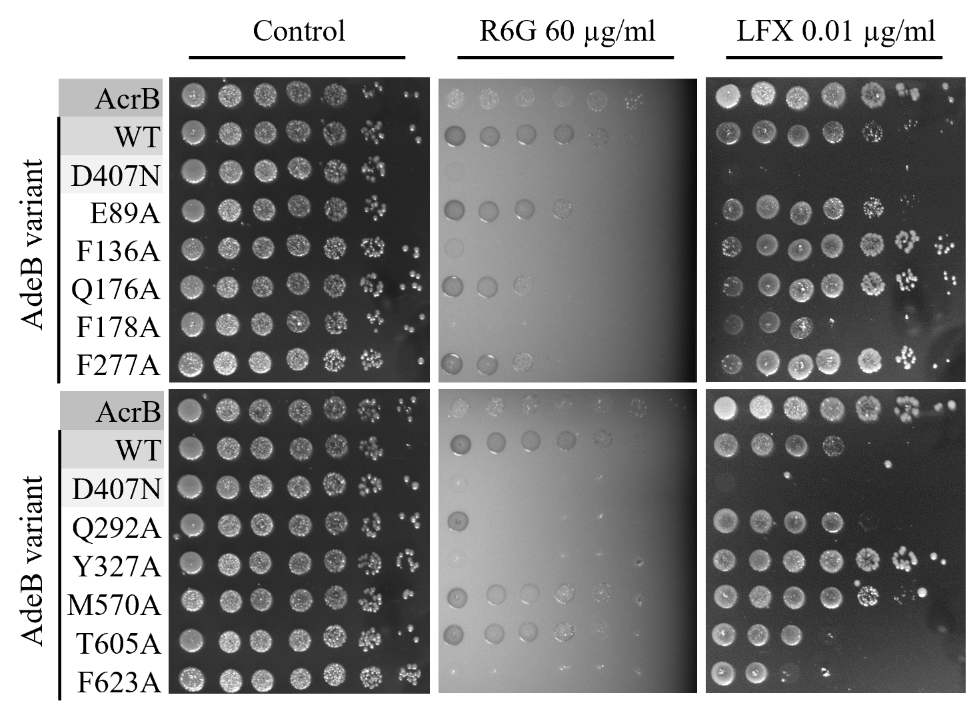


Replicate 3


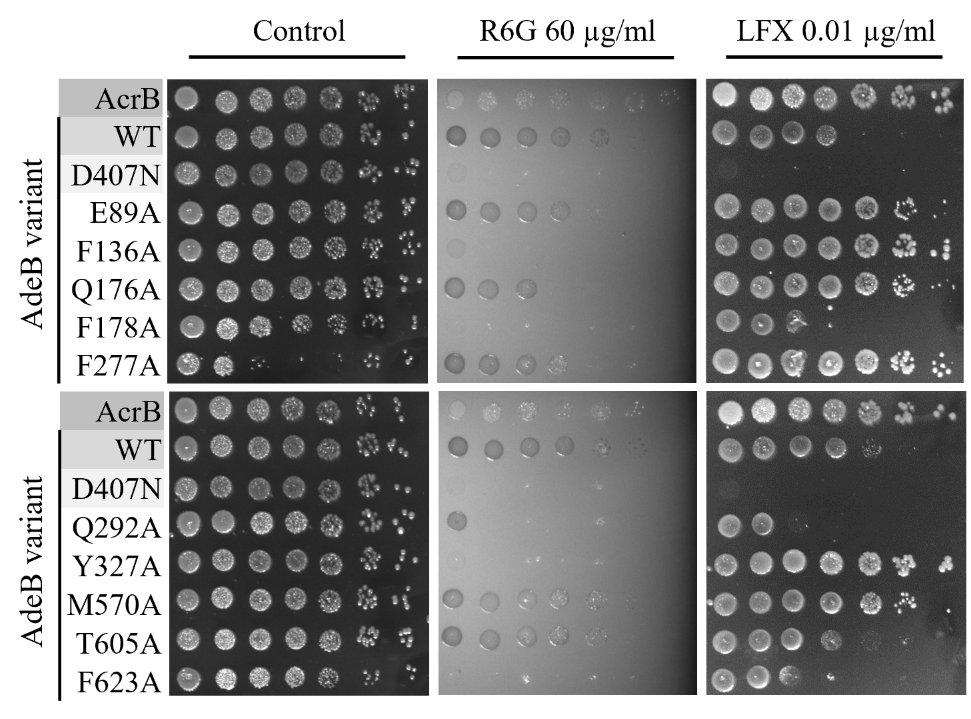


1. **Drug agar plate dilution assays**

Replicate 1


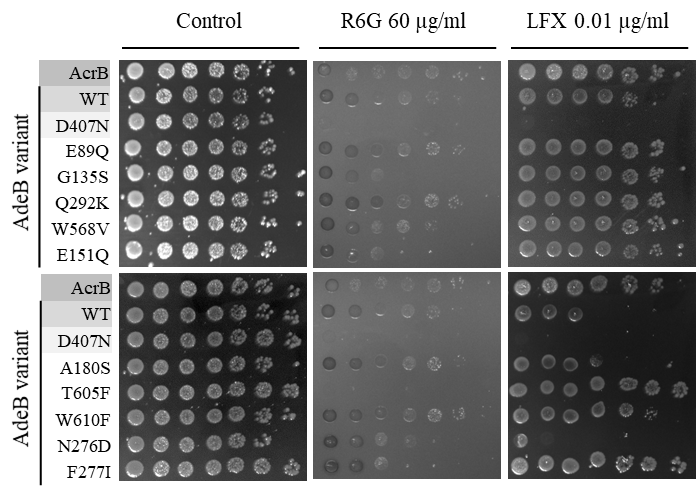


Replicate 2


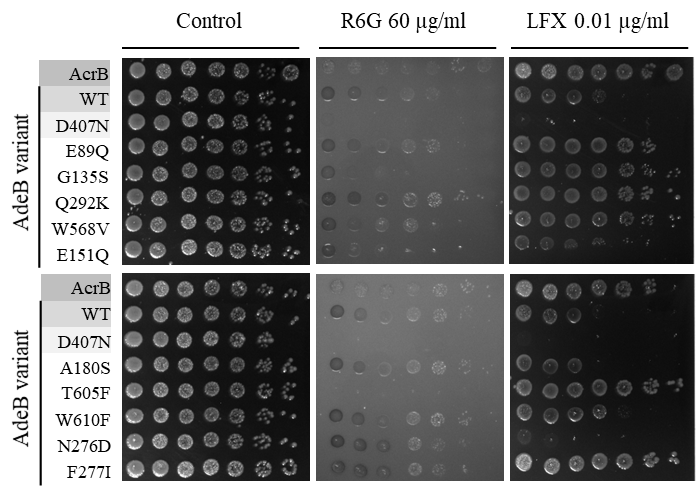


Replicate 3


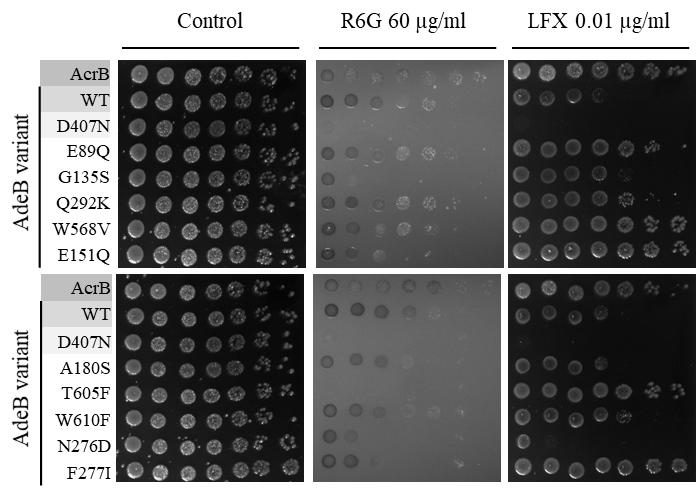


1. **Western Blot with anti-His-antibody to detect AdeB variants and AcrB (whole cells)**


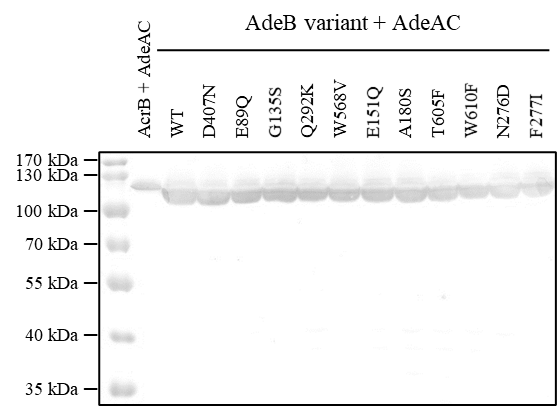


**Source data of supplementary figure 15**

1. **Ethidium accumulation**

Replicate 1

**
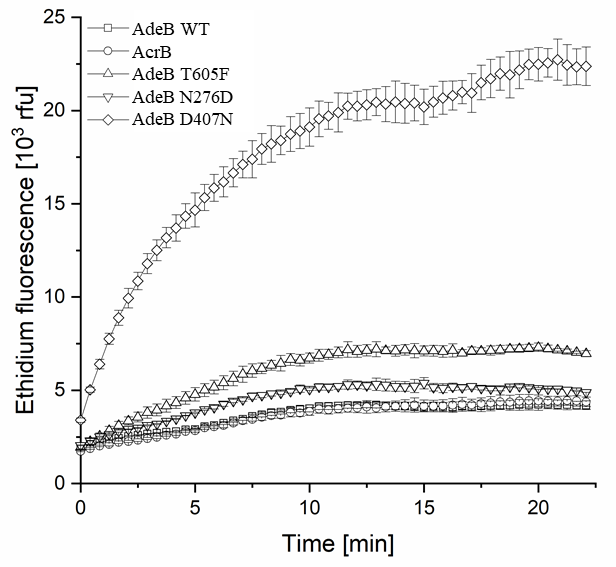
**

Replicate 2


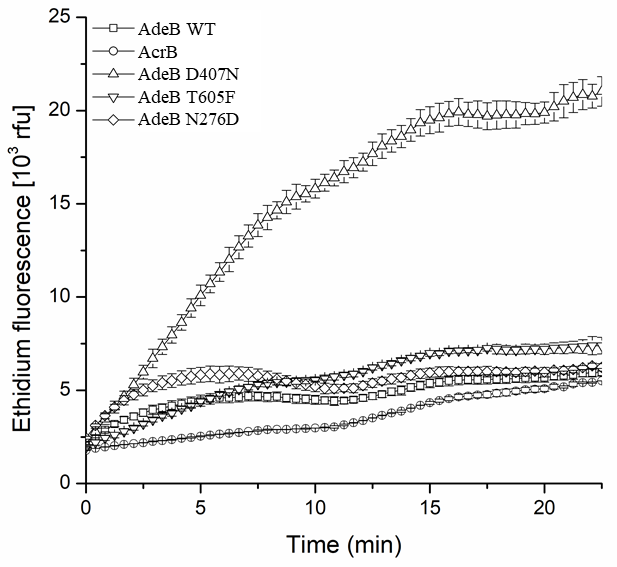


Replicate 3


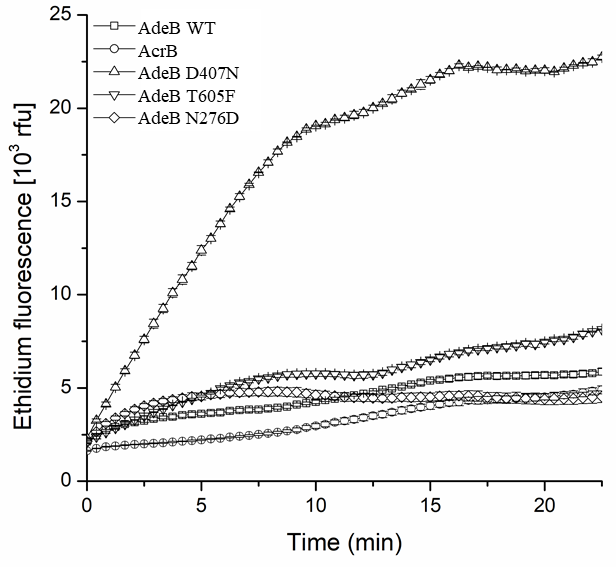


1. **Western Blot with anti-His-antibody to detect AdeB variants and AcrB; anti-Myc-antibody to detect AdeA; anti-Strep-antibody to detect AdeC (whole cells)**

**
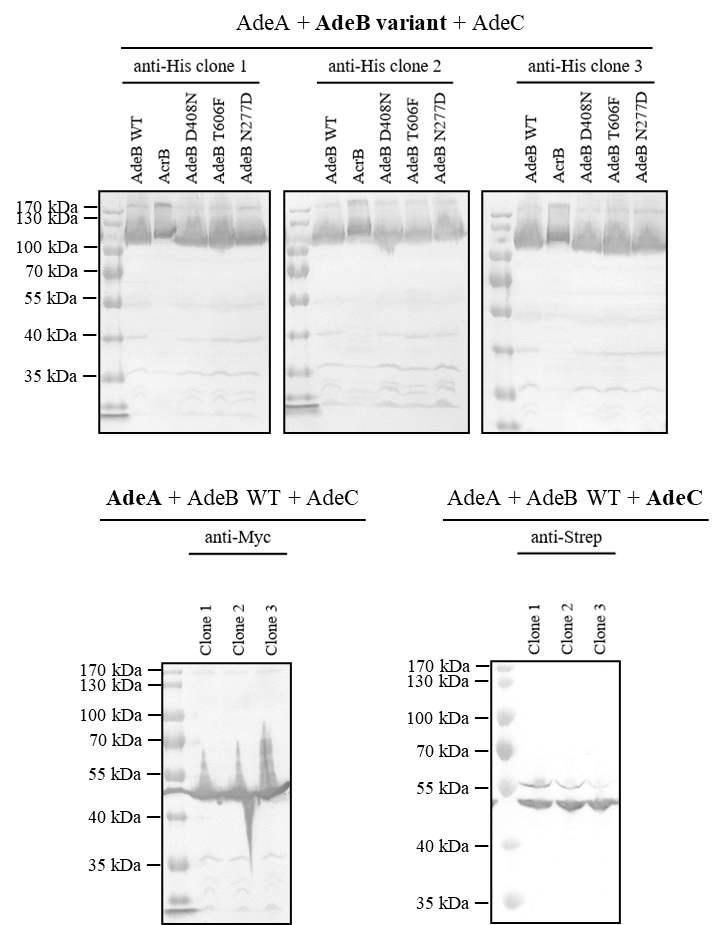
**
